# Benefits and harms of Risperidone and Paliperidone for treatment of patients with schizophrenia or bipolar disorder: a meta-analysis involving individual participant data and clinical study reports

**DOI:** 10.1186/s12916-021-02062-w

**Published:** 2021-08-25

**Authors:** Alexander Hodkinson, Carl Heneghan, Kamal R. Mahtani, Evangelos Kontopantelis, Maria Panagioti

**Affiliations:** 1 National Institute for Health Research School for Primary Care Research, Centre for Primary Care and Health Services Research, Division of Population Health, Health Services Research and Primary Care, School of Health Sciences, Faculty of Biology, Medicine and Health, Manchester Academic Health Science Centre, University of Manchester, Williamson Building, Oxford Road, Manchester, M13 9PL UK; 2 National Institute for Health Research Greater Manchester Patient Safety Translational Research Centre, School of Health Sciences, University of Manchester, Manchester, M13 9PL UK; 3 Nuffield Department of Primary Care health Sciences, University of Oxford, Oxford, UK; 4 Division of Informatics, Imaging & Data Sciences, University of Manchester, Manchester, M13 9PL UK

**Keywords:** Antipsychotics, Risperidone, Paliperidone, Schizophrenia, Bipolar disorder, Individual participant data, Meta-analysis, Clinical study reports

## Abstract

**Background:**

Schizophrenia and bipolar disorder are severe mental illnesses which are highly prevalent worldwide. Risperidone and Paliperidone are treatments for either illnesses, but their efficacy compared to other antipsychotics and growing reports of hormonal imbalances continue to raise concerns*.* As existing evidence on both antipsychotics are solely based on aggregate data, we aimed to assess the benefits and harms of Risperidone and Paliperidone in the treatment of patients with schizophrenia or bipolar disorder, using individual participant data (IPD), clinical study reports (CSRs) and publicly available sources (journal publications and trial registries).

**Methods:**

We searched MEDLINE, Central, EMBASE and PsycINFO until December 2020 for randomised placebo-controlled trials of Risperidone, Paliperidone or Paliperidone palmitate in patients with schizophrenia or bipolar disorder. We obtained IPD and CSRs from the Yale University Open Data Access project. The primary outcome Positive and Negative Syndrome Scale (PANSS) score was analysed using one-stage IPD meta-analysis. Random-effect meta-analysis of harm outcomes involved methods for coping with rare events. Effect-sizes were compared across all available data sources using the ratio of means or relative risk. We registered our review on PROSPERO, CRD42019140556.

**Results:**

Of the 35 studies, IPD meta-analysis involving 22 (63%) studies showed a significant clinical reduction in the PANSS in patients receiving Risperidone (mean difference − 5.83, 95% CI − 10.79 to − 0.87, *I*^2^ = 8.5%, *n* = 4 studies, 1131 participants), Paliperidone (− 6.01, 95% CI − 8.7 to − 3.32, *I*^2^ = 4.3%, *n* = 13, 3821) and Paliperidone palmitate (− 7.89, 95% CI − 12.1 to − 3.69, *I*^2^ = 2.9%, *n* = 5, 2209). CSRs reported nearly two times more adverse events (4434 vs. 2296 publication, relative difference (RD) = 1.93, 95% CI 1.86 to 2.00) and almost 8 times more serious adverse events (650 vs. 82; RD = 7.93, 95% CI 6.32 to 9.95) than the journal publications. Meta-analyses of individual harms from CSRs revealed a significant increased risk among several outcomes including extrapyramidal disorder, tardive dyskinesia and increased weight. But the ratio of relative risk between the different data sources was not significant. Three treatment-related gynecomastia events occurred, and these were considered mild to moderate in severity.

**Conclusion:**

IPD meta-analysis conclude that Risperidone and Paliperidone antipsychotics had a small beneficial effect on reducing PANSS score over 9 weeks, which is more conservative than estimates from reviews based on journal publications. CSRs also contained significantly more data on harms that were unavailable in journal publications or trial registries. Sharing of IPD and CSRs are necessary when performing meta-analysis on the efficacy and safety of antipsychotics.

**Supplementary Information:**

The online version contains supplementary material available at 10.1186/s12916-021-02062-w.

## Background

Schizophrenia and bipolar disorder are highly prevalent and debilitating severe mental illnesses *worldwide.* Patients often experience both simultaneously because they share a similar causative process of diagnoses [1].

Risperidone is a leading second-generation antipsychotic drug approved for the treatment of schizophrenia in adults and adolescents and for the short-term treatment of manic or mixed episodes of bipolar disorder [2, 3]. It is indexed on the World Health Organization’s List of Essential Medicines as one of the most effective and safe medicines [4]. However, the manufacturers ‘Johnson & Johnson’ have been involved in over 13,500 legal (lawsuit) cases because of their failure to disclose that Risperidone may cause hormonal imbalances that could lead to breast tissue development (‘gynecomastia’) and increased blood prolactin levels (‘galactorrhoea hyperprolactinaemia’) in boys and girls [5, 6]. There have also been ‘black box warnings’ of misleading marketing of the drug for off label uses in children and adolescents with schizophrenia or bipolar disorder and in elderly patients with dementia [7, 8]. Moreover, the clinical benefit of Risperidone is questionable when compared to other antipsychotics and was recently found to be only the sixth best treatment option for overall change in symptoms of schizophrenia [9, 10].

Paliperidone is another second-generation antipsychotic drug also manufactured by Johnson & Johnson and used to treat schizophrenia. As both Risperidone and Paliperidone act via the same pathways in the body, research have suggested strong links of drug-induced hormonal imbalances in both antipsychotics [11], and like Risperidone, the efficacy of Paliperidone over other antipsychotics is also questionable [10]. Other serious adverse events experienced by patients with severe mental illnesses receiving either Risperidone or Paliperidone include muscle-related ‘extrapyramidal effects’; permanent movement disorders such as ‘tardive dyskinesia’; cerebrovascular events including stroke, transient ischaemic attack, vascular malformation and venous thromboembolism [12, 13]; and neuroleptic malignant syndrome, increased risk of suicide and weight gain [14, 15]. However, these harms are rarely reported exhaustively in journal publications.

The existing evidence that support policy on both antipsychotic drugs is solely based on aggregate data meta-analyses of published randomised controlled trials (RCTs) involving mostly adults [16]. Analyses of more exhaustive forms of unpublished data including individual participant data (IPD) and clinical study reports (CSRs) are increasingly recommended for evaluating the full evidence base for the effectiveness and safety of these drugs. Such analyses can create clear hierarchies of evidence about the benefit and harms of both interventions, taking into account reporting bias, and have the potential to better inform policy decisions.

The Yale University Open Data (YODA) project was setup to promote better transparency of clinical trial results. As of 2014, YODA have been enabling scientists across the world to gain access to Johnson & Johnsons clinical trial data assets including Risperidone and Paliperidone [17–19]. Using more innovative methodologies on a hitherto unavailable and more complete database from IPD and CSRs contained in YODA, we aim to compare in a meta-analysis the benefit and harms of Risperidone and Paliperidone with data from corresponding trial register entries and journal publications.

## Methods

The study followed a registered (PROSPERO CRD42019140556 [20]) protocol and findings are reported in accordance with the PRISMA-IPD statement [21].

### Search methods

Searches were done from inception until December 2020 without language restriction in the MEDLINE (Ovid), Cochrane Central Register of Controlled Trials, EMBASE (*EBSCO)* and PsycINFO (see searches in Additional file 1: Table S1). Key terms used in the searches were intervention-related (Risperidone, Risperdal, Risperdal Consta, Paliperidone, Invega, Trevicta and Xeplion) and condition-related (schizophrenia, psychosis, psychotic, bipolar disorder and cyclothymic disorder). The number of citations identified varied between database with EMBASE identifying 361 citations and MEDLINE 51 citations. Trial registers including ClinicalTrials.gov, the WHO ICTRP portal and OpenTrials.net and drug approval packages at the Food and Drug Administration [22] and European Product Assessment Reports [23] were searched for further studies.

### Eligibility

We included randomised placebo-controlled trials involving patients diagnosed with schizophrenia or bipolar disorder.

Interventions include Risperidone (brand name: Risperdal (oral) and Risperdal Consta (intravenously injected)), Paliperidone (brand name: Invega and Trevicta) or Paliperidone palmitate (brand name: Xeplion) with any form of application at any dose. As the included trials were placebo-controlled, the comparator was therefore a placebo pill or kit vials which contained microspheres with no active drug.

The primary outcome for efficacy includes the Positive and Negative Syndrome Scale (PANSS) total score comprising the three subscales positive, negative and general psychopathology which are regarded as the ‘gold standard’ for assessment of psychotic behaviour disorders. We used a 30-point subtraction to calculate the PANSS total score which is important for interpreting the percentage change of improvement in the mental health state of the patients [24]. Based on previous recommendations [25], we categorised the difference in PANSS from baseline using following improvement thresholds: 25% (minimal improvement), 50% (good clinical response) and 75%. Secondary outcomes for efficacy include the mean time after treatment until relapse, Clinical Global Impression-Severity (CGI-S) scale and the Young Mania Rating Scale (YMRS).

For safety, primary outcomes include treatment-emergent adverse events (AEs), serious adverse events (SAEs) following the Food and Drug Administration’s definition (including life-threatening, hospitalisation, disability or permanent damage, congenital anomaly/birth defect, required intervention to prevent permanent impairment or damage or other serious important medical events), discontinuations due to AE, gynecomastia and drug-induced death. Secondary outcomes include cerebrovascular events, extrapyramidal disorder, weight increased, tardive dyskinesia and behaviour-related outcomes (i.e., aggression, irritability and intentional self-injury). These outcomes were informed by previous meta-analyses [10, 26–28], patient and public involvement feedback and black box warnings [29].

### Data collection, extraction and risk of bias

A research application was made for IPD and CSRs of each of the publications identified at YODA [30]. We were able to access another trial [31] upon request which was not listed on their website; but trials not sponsored by Johnson & Johnson could not be provided [32–34]. Further requests for CSRs were made at the European Medicines Agency for the two centrally licenced antipsychotics Paliperidone and Paliperidone palmitate. We were successful in retrieving two CSRs [35, 36] for Paliperidone at the European Medicines Agency clinical data website [37]. But, because of an ongoing court case about public access to clinical trial data from 2019 to 2020 [38, 39], this meant that we were unable to obtain access to further trial CSRs.

Data extractions were conducted by two independent reviewers (AH, MP). Discrepancies were resolved through consensus or recourse to a third reviewer (EK). For each matching document pair (CSR, Registry report and Journal publication) supplemented by the IPD; the study characteristics, content and a comparison of reporting were done using the criteria as outlined in Additional file 2: Table S2. Risk of bias for each study was assessed by two reviewers (AH, MP) using the Cochrane Risk of Bias (RoB) tool [40]. In each domain, the scores of the RoB assessment reflected all the available evidence and thus allowed for downgrading of RoB by considering any new evidence available from the corresponding CSR that are not reported in the journal publication.

### Data synthesis

In this comparison, pooled estimates for all outcomes of interest were compared across the three data sources (CSR, trial registry and journal publication) to assess for consistency. The pooled effects from each data source were then compared using the ‘ratio of relative risks’ or ‘ratio of means’ to observe for statistical differences [41]. For the primary efficacy outcome (PANSS), IPD-MAs effect-sizes were included also in the ratio of mean comparisons. In total, sixty-one possible outcome-specific meta-analysis comparisons were made enabling comparisons of the pooled effects from the different data sources.

Aggregate data from CSRs, journal publication and registry reports were meta-analysed using DerSimonian-Laird random-effects [42]. For continuous outcomes, standardised mean differences (SMD) were calculated using Hedges’ *g* [43] and interpreted according to Cohen’s criteria [44]. Hartung-Knapp confidence intervals were used to account for uncertainty in the variance estimate [45]. For dichotomous outcomes (including AEs), effects were assessed by pooling the relative risk (RR). Effect estimates were pooled across trials using Mantel Haenszel fixed-effect or inverse-variance random-effects meta-analysis dependent upon the number of studies reporting the outcome of interest. A sensitivity analysis was also performed pooling the relative difference instead of RR for rare events [46, 47]. For studies reporting single or double zero events in one or both treatment group, we used the ‘exact’ fixed-effect meta-analysis method for pooling the effects [48]. Safety narratives were also obtained from the listings data where possible.

We then performed the one-stage IPD meta-analysis for the primary outcome PANSS total score [49, 50]. Scores were adjusted ensuring zero as the lowest possible score [51]. Meta-analysis of the non-standardised PANSS total score was used as the primary measure to assess the percentage reduction change for clinical improvement. Missing data were imputed using the R package ‘MICE: Multivariate Imputation by Chained Equations’ following Rubin’s rules [52]. The imputed values used observed values of the primary outcome PANSS and baseline covariates (study, interventions, age, gender and baseline PANSS score) to predict missing data. Convergence of the algorithms was assessed, and sensitivity analyses were performed using only cases with available data.

The analyses used ‘one-stage’ linear mixed effect models which incorporated random-effects to allow for heterogeneity across trials [50, 53, 54], fitted using Stata (software version 16.1) [55] command *mixed* and the *ipdforest* command to summarise the evidence by study and obtain forest plots [56]. Restricted maximum likelihood was used for model estimation and centring of covariates by study-specific means was performed to avoid aggregation bias [57]. Differential effects were investigated by adding covariate parameters (age, gender and ethnicity) and interactions between covariate (treatment-covariate interaction terms) and antipsychotic treatment to the linear mixed model [58].

We used the *I*^2^ statistic to determine the magnitude of heterogeneity and associated 95% confidence intervals (CIs) [59, 60]. Effects across different type of severe mental illness and dosage regiments (approved according to the national US [29, 61, 62] and EU guidelines [63, 64] vs. other doses) were assessed in subgroup analyses. Sensitivity analysis of the effects of just including low RoB studies was performed for the PANSS outcome.

### Patient and public involvement

A group of 31 partners who were members of an established patient and public involvement group were consulted about the appropriateness of our research questions, and selection of the outcome measures of this study. A patient representative provided input to the interpretation and writing up of results. The dissemination plan targets a wide audience, including members of the public, patients, health professionals and experts in the specialty through various channels: written communication, events and conferences, networks and social media.

## Results

**Fig. 1:**
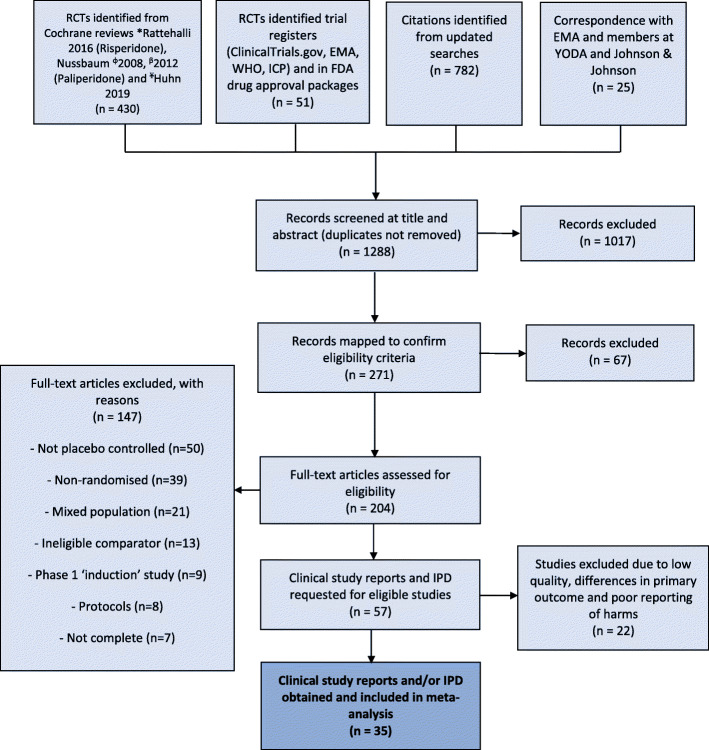
PRISMA flowchart of search strategy showing trials identified through literature search and previous meta-analyses, trial registers and through correspondence with YODA and regulators. RCTs, randomised controlled trials; EMA, European Medicines Agency; FDA, Food and Drug Administration; WHO, World Health Organisation; YODA, Yale Open Data Access. Asterisk indicates [27]; phi symbol, [28]; beta symbol, [65]; and Yen symbol [10]

A total of 1288 references were retrieved. Following full-text screening of 204 studies, 57 RCTs met our inclusion criteria and IPD/CSRs were accessible for 35 (61%) studies involving 12,316 patients (Fig. 1). The characteristics of the 35 studies are provided in Additional file 3: Table S3 [31, 35, 36, 66–96]. The remaining 22 studies were excluded due to their low quality, differences in primary outcome selection with only a single subscale being used to measure PANSS and a general poor reporting of harms meaning data could not be include in the meta-analysis.

### Characteristics of included studies

Twenty-five studies (71%) were carried out in America and seven (20%) in Europe. The median number of patients across the studies was 323 (IQR, 263). The median age across studies was 39 (IQR, 4) years, and only three studies (9%) [66, 67, 97] involved children or adolescents under the age of 19 years. Twenty-eight of the studies (80%) involved more male patients than female.

Twenty-five (71%) of the studies involved patients with schizophrenia, and 10 (29%) involved patients with bipolar disorder. Paliperidone was administered in sixteen (46%) of the studies comprising 4920 participants, eleven studies (31%; 3697 participants) used Risperidone (six involving oral administered Risperidone and five intravenous administration of Risperidone Consta) and eight studies (23%; 3699 participants) used Paliperidone palmitate. Oral and intravenously administered Risperidone were combined into one analysis due to the limited number of studies available in the IPD meta-analysis. The median length of the intervention was 63 (IQR, 53) days. The doses used for oral administration of Risperidone and Paliperidone ranged from 1 to 75 mg and for long-acting injectable Paliperidone palmitate, 25 to 150 mg.

### Description of data

Full IPD data including demographic, efficacy and safety listings data were available for 34 (97%) of the studies.

The 35 CSRs comprised of a median 825 (IQR, 548) pages (Additional file 4: Table S4). Statistical analysis plans and protocols were provided separately for 32 (91%) of the CSRs. A low level of redactions in the CSRs (i.e. subject ID and investigator names) were found in only three of the studies [67–69], 29 studies presented with a medium level of redactions (i.e. narratives removed from the appendix) and three [70, 71, 98] had a high level of redactions (i.e. pages removed from the core report and/or some outcome data were redacted) (Additional file 5: Table S5). Only adverse events with over 5% incidence in any treatment group were reported in 22 of the CSRs (63%), and the remaining 13 (37%) studies reported only AEs with over 2% incidence.

### Quality assessment of the studies

Risk of bias assessment of the studies involving all data sources combined, is shown in Additional file 6: Table S6. For Risperidone, one study had overall high RoB, four had a moderate RoB and six with low RoB. For Paliperidone, two studies scored with a high RoB, five with moderate RoB and 10 with low RoB. Seven Paliperidone palmitate studies scored with low RoB and only one study scored with a moderate RoB. The domain for ‘Blinding of participants and personnel’ had the greatest number of studies (*n* = 4) at high risk. CSRs were responsible for downgrading individual high-risk study domains to low on 36% of occasions.

### Comparison of reporting of design factors and outcomes between sources at study level

Compared to the trial register entries and journal publications, the CSRs reported on average more design factors including randomisation (86% vs. 0% vs. 64%, respectively), allocation concealment (80% vs. 3% vs. 33%) and blinding (100% vs. 6% vs. 58%) (Table 1). Sample size determination was reported in 91% of CSRs, compared to 52% of journal publications and 3% of registry reports.

**Table 1:** Comparison of reporting of design factors, statistical analysis and reporting of efficacy and safety outcomes across all three data sources

Key information based on reporting of design aspects and statistical analysis, and reporting of efficacy and safety sections	CSR (***N*** = 35)	Trial registry (***N*** = 34)	Journal Publication(s) (***N*** = 33)
*Method and design*			
Randomisation	30 (86%)	0 (0%)	21 (64%)
Allocation concealment	28 (80%)	1 (3%)	11 (33%)
Blinding	35 (100%)	2 (6%)	19 (58%)
Sample size calculation	32 (91%)	1 (3%)	17 (52%)
Inclusion criteria specified	35 (100%)	32 (94%)	32 (97%)
Definition of causality provided	30 (86%)	1 (3%)	5 (15%)
Intervention and comparator			
Intervention used	35 (100%)	30 (88%)	33 (100%)
Dose and delivery	35 (100%)	10 (29%)	27 (82%)
Placebo pill explained (i.e. visual detail)	27 (77%)	1 (3%)	9 (27%)
*Outcomes*			
Primary outcome specified	35 (100%)	30 (88%)	25 (76%)
Secondary outcomes specified	35 (100%)	29 (85%)	20 (61%)
HRQoL measured (i.e. SQLS or sleep quality)	26 (74%)	3 (9%)	9 (27%)
Symptoms (depression or suicidality)	34 (97%)	1 (3%)	5 (15%)
*Safety*			
AEs	35 (100%)	10 (29%)	29 (88%)
SAEs	35 (100%)	17 (49%)	20 (61%)
Discontinuation due to AEs	33 (94%)	9 (26%)	20 (61%)
Reason for withdrawal	31 (89%)	1 (3%)	11 (33%)
Death	35 (100%)	9 (26%)	29 (88%)
Reason for death	35 (100%)	33 (97%)	30 (91%)

*CSR* clinical study reports, *HRQoL* health-related quality of life, *SQLS* sleep quality of life survey, *AEs* adverse events, *SAEs* serious adverse events

Primary and secondary outcomes were reported with 100% consistency in the CSRs, 88% and 85% in the registry reports and 76% and 61% in the journal publications. Clear narratives for deaths were reported in 88% of journal publications compared to CSRs. Reporting of AEs was poor in registry reports with only 29% of studies providing amenable/extractable data. Reporting of SAEs was also poor in both journal publications and registry reports, with only 61% and 49% of studies providing data. In contrast AEs and SAEs were reported more clearly in the CSRs of each study, as were discontinuations.

### Data synthesis of efficacy outcomes

**Fig. 2:**
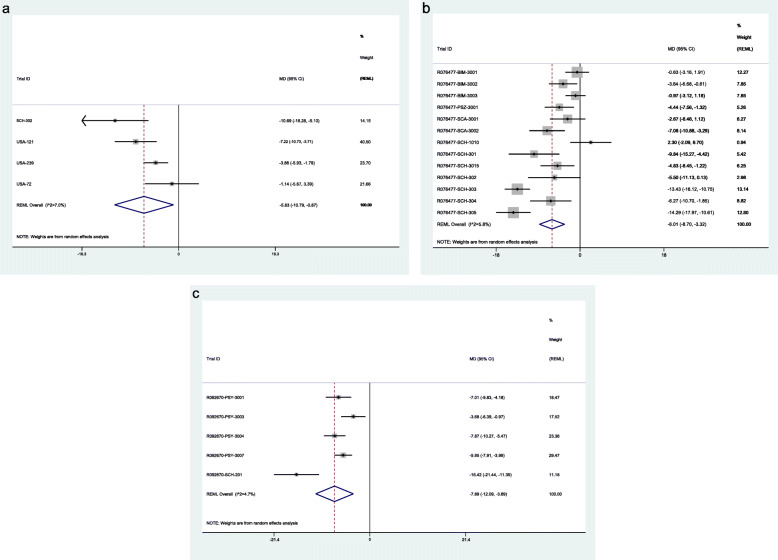
**a** One-stage IPD meta-analysis of Risperidone efficacy according to total PANSS. MD, mean difference; 95% CI, 95% confidence interval; REML, restricted maximum likelihood. **b** One-stage IPD meta-analysis of Paliperidone efficacy according to total PANSS. MD, mean difference; 95% CI, 95% confidence interval; REML, restricted maximum likelihood. **c** One-stage IPD meta-analysis of Paliperidone palmitate efficacy according to total PANSS. MD, mean difference; 95% CI, 95% confidence interval; REML, restricted maximum likelihood

IPD meta-analysis involving 7161 patients from 22 studies enabling calculation of the PANSS total score, showed a statistically significant clinical reduction of PANSS in participants receiving Risperidone (mean difference − 5.83, 95% CI − 10.79 to − 0.87 [SMD = − 0.29, 95% CI − 0.55 to − 0.03], *I*^2^ = 8.5 (1.6 to 43.2) %, *n* = 4 studies, 1131 participants) (Fig. 2a), Paliperidone (− 6.01, − 8.7 to − 3.32 [SMD = − 0.29, 95% CI − 0.40 to − 0.17], *I*^2^ = 4.3 (1.5 to 12.1) %, *n* = 13, 3821) (Fig. 2b) and Paliperidone palmitate (− 7.89, − 12.1 to − 3.69 [SMD = − 0.35, 95% CI − 0.50 to − 0.19], *I*^2^ = 2.9 (0.4 to 20.2) %, *n* = 5, 2209) (Fig. 2c). No significant covariate interactions were found in age, gender or ethnicity (see Additional file 7: Table S7).

Meta-analyses of the PANSS total score and the secondary efficacy outcomes in CSR, journal publications and registry reports were analysed separately and are provided in Additional file 8: Table S8.

A comparison of the ratio of means for the pooled PANSS total score across all four data sources are reported in Table 2. No significant differences were observed across all three interventions. Similarly, the effect measures for the secondary efficacy outcomes led to no significant differences when comparing the ratios of the pooled effect estimates between the different data sources (see Table 3).

**Table 2:** Ratio of means with 95% CIs of PANSS total scores compared between the four sources of data (individual participant data, clinical study report, journal publication and registry report)

Treatment	IPD vs. CSR	IPD vs. journal publication	IPD vs. registry report	CSR vs. journal publication	CSR vs. registry report	Journal publication vs. registry report
**Risperidone**	0.87 (− 0.37, 1.48)	0.21 (− 0.05, 0.68)	NA	0.38 (− 0.07, 0.81)	NA	NA
**Paliperidone**	0.29 (− 0.02, 0.60)	0.11 (− 0.17, 0.87)	0.49 (− 0.66, 1.24)	0.49 (− 0.39, 1.37)	0 (− 0.99, 0.99)	− 0.49 (− 1.75, 0.78)
**Paliperidone palmitate**	0.11 (− 0.13, 0.29)	0.08 (− 0.17, 0.33)	0.21 (− 0.53, 0.95)	0 (− 0.23, 0.23)	0.13 (− 0.22, 0.48)	0.13 (− 0.25, 0.51)

*IPD* individual participant data, *CSR* clinical study report, *NA* not applicable

**Table 3:** Ratio of risks, odds, difference or means of secondary efficacy outcomes and harm outcomes across the three sources of data (clinical study report, journal publication and registry report)

Outcomes	CSR vs. journal publication	CSR vs. registry report	Journal publication vs. registry report
**Risperidone**			
*Efficacy*			
Time to relapse	ROR = 1.43 (0.31, 6.48)	ROR = 1.73 (0.39, 7.65)	ROR = 1.21 (0.56, 2.61)
YMRS	ROM = − 1.83 (− 4.76, 1.10)	ROM = − 2.38 (− 4.64, 0.12)	ROM = − 2.38 (− 4.64, 0.12)
CGI-S	ROM = − 0.56 (− 3.38, 2.26)	NA	NA
*Safety*			
TEAEs	RRR = 0.97 (0.83, 1.14)	RRR = 1.03 (0.86, 1.23)	RRR = 1.06 (0.84, 1.33)
SAEs	RRR = 0.70 (0.14, 3.42)	RRR = 1.48 (0.64, 3.45)	RRR = 2.13 (0.37, 12.3)
Discontinuation due to AEs	RRR = 1.14 (0.63, 2.05)	RRR = 0.65 (0.16, 2.67)	RRR = 0.57 (0.14, 2.42)
Death	NA	NA	NA
**Paliperidone**			
*Efficacy*			
Time to relapse	ROR = 3.63 (0.33, 39.44)	ROR = 0.31 (0.03, 3.84)	ROR = 0.09 (0.03, 0.27)
YMRS	ROM = 0.96 (− 1.73, 3.65)	ROM = − 0.28 (− 1.93, 1.37)	ROM = − 1.24 (− 4.16, 1.68)
CGI-S	ROM = 2 (− 1.13, 5.13)	ROM = − 0.46 (− 1.83, 0.91)	ROM = − 2.46 (− 5.52, 0.60)
*Safety*			
TEAEs	RRR = 1 (0.91, 1.10)	RRR = 0.86 (0.71, 1.03)	RRR = 0.86 (0.71, 1.04)
SAEs	RRR = 1 (0.61, 1.64)	RRR = 1.38 (0.40, 4.84)	RRR = 1.38 (0.37, 5.17)
Discontinuation due to AEs	RRR = 1 (0.55, 1.80)	RRR = 1.04 (0.36, 3.00)	RRR = 1.04 (0.37, 2.89)
Death	NA	NA	NA
**Paliperidone palmitate**			
*Efficacy*			
Time to relapse	ROR = 1.25 (0.03, 60.54)	ROR = 0.17 (0.003, 8.75)	ROR = 0.14 (0.06, 0.30)
YMRS	NA	NA	NA
CGI-S	NA	ROM = 0 (− 0.32, 0.32)	NA
*Safety*			
TEAEs	RRR = 0.95 (0.79, 1.15)	RRR = 0.90 (0.76, 1.07)	RRR = 0.95 (0.74, 1.21)
SAEs	RRR = 2.54 (0.81, 7.98)	RRR = 1.13 (0.62, 2.05)	RRR = 0.44 (0.13, 1.47)
Discontinuation due to AEs	RRR = 0.88 (0.24, 3.20)	RRR = 0.96 (0.47, 1.99)	RRR = 1.09 (0.34, 3.45)
Death	NA	NA	NA

*ROM* ratio of means, *RRR* ratio of relative risks, *ROR* ratio of odds ratio, *NA* not applicable, *CSR* clinical study report, *PANSS* Positive and Negative Syndrome Scale, *YMRS* Young Mania Rating Scale, *CGI-S* Clinical Global Impression rating scales, *TEAEs* treatment-emergent adverse events, *SAEs* serious adverse events

### Reporting and data synthesis of harm outcomes

**Fig. 3:**
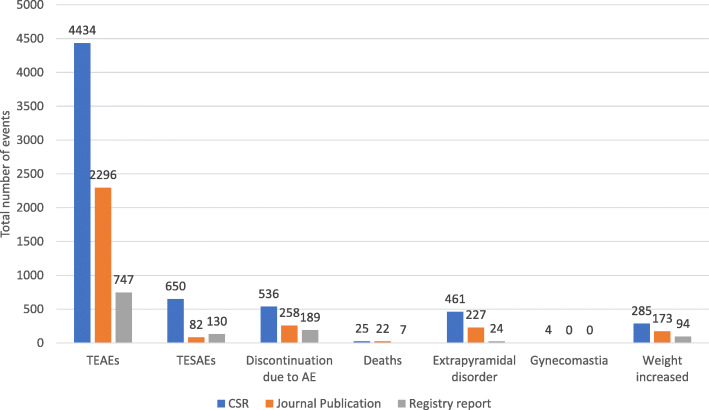
Display of the total number of events combined over the 35 studies for key safety outcomes by the source of data. See ‘Additional file 11: Table S10’ for sensitivity analysis for causes of deaths and gynecomastia cases and the forest plots in ‘Additional file 14: Fig S2’ provide the total pooled relative risks/risk difference estimates. ‘Additional file 9: Table S9’ provides the relative differences in reporting and the ratio of relative risks for TEAEs and TESAEs. TEAEs, treatment-emergent adverse events; TESAEs, treatment-emergent serious adverse events; AE, adverse event; CSR, clinical study report

The reported harm outcomes across the three different sources of data are presented in Fig. 3.

AEs were reported nearly twice as often in CSRs compared to journal publications (4434 vs. 2296, showing a relative difference (RD) = 1.93, 95% CI 1.86 to 2.00) and by almost six-fold compared to registry reports (4434 vs. 747 showing RD = 5.94, 95% CI 5.54 to 6.36). Similarly, SAEs were reported almost eight times and five times more often in CSRs compared to the journal publications (650/82; RD = 7.93, 95% CI 6.32 to 9.95) and registry reports (650/130; RD = 5, 95% CI 4.16 to 6.02), respectively. However, the ratio of relative risks for AEs and SAEs comparing CSRs with journal publications or registry reports were not significantly different (Additional file 9: Table S9).

Study discontinuations caused by AE (536/258), extrapyramidal disorders (461/227) and increase weight (285/173) were all reported nearly twice as often in the CSRs compared to journal publications. Drug-induced deaths and gynecomastia were consistently reported between CSRs and publications. Reporting rates for all the harm outcomes are provided at study level in Additional file 10: Fig S1.

Patient safety narratives revealed that the cause of drug-induced deaths was due to suicide (depression or overdose), coronary heart disease and stroke. The three cases of gynecomastia in treatment group were reported only in adults as being probably related to treatment but were considered mild to moderate in nature (see narratives in Additional file 11: Table S10).

Results of all harm outcomes by each data source and intervention are presented in Additional file 8: Table S8. There was a significant increased risk of extrapyramidal disorders and increased weight in all three interventions when pooling across the CSRs. Overall, the risk of an AE increased whilst receiving Risperidone or Paliperidone. However, the ratio of relative risks or risk differences of the pooled effects between the different data sources, showed no significant differences (Table 3).

### Subgroup analyses

A significant increased risk of AEs, SAEs or extrapyramidal disorders was found in schizophrenia diagnosed patients when pooled across the CSRs (Additional file 12: Table S11). The pooled effects from journal publications for these same outcomes did not show an increase in risk. Risperidone when provided at a dose above 6 mg showed a significant reduction in PANSS score when based on data from the journal publications. However, no significant reduction was found in the pooled effects using data from the CSRs (Additional file 13: Table S12). Forest plots for all meta-analysis are provided in Additional file 14: Fig S2. Sensitivity analysis involving only low risk of bias studies did not reveal any significant differences in PANSS score (Additional file 15: Fig S3).

## Discussion

This meta-analysis presents the most in-depth investigation to date of the efficacy and safety of Risperidone and Paliperidone in patients with schizophrenia or bipolar disorder.

Our IPD meta-analysis showed a significant reduction in the PANSS total scores for the use of both Risperidone and Paliperidone. However, the effects were more conservative when compared with the results of a recent network meta-analysis involving both oral forms of interventions compared against placebo (i.e., Risperidone: SMD = − 0.55 vs. − 0.32; Paliperidone: SMD = − 0.49 vs. − 0.31) [10]. More importantly, the actual clinical reduction in the PANSS total score was smaller than the evidence reported in three separate Cochrane reviews comparing each intervention to placebo (Risperidone − 17.81 (− 18.17, − 17.45) [27] vs. − 5.83 (− 10.79 to − 0.87); Paliperidone − 8.99 (− 11.1, − 6.9) [28] vs. − 6.01 (− 8.7 to − 3.32); Paliperidone palmitate − 8.07 (− 9.75, − 6.39) [65] vs. − 7.89 (− 12.1 to − 3.69)). Given that our estimates are based on a similar number of patients, these differences could be attributed to methodological limitations of these previous studies compared to our more in-depth and robust analysis approach. Previous meta-analyses have relied upon data from published literature of these three interventions which make them more vulnerable to reporting biases [99] and are overly reliant on treatment effects that have been estimated using less robust methods.

Our most notable finding was the inconsistencies in the reporting of harms between CSRs and journal publications. CSRs provided over double the number of AEs, and almost eight times more SAEs showing a significant increase in the relative differences favouring CSRs. The effect estimates from CSRs which involved more patients and studies, revealed an increased risk in the number of AEs and elevated risks for other harms such as ‘extrapyramidal disorder’, ‘tardive dyskinesia’ and ‘weight increased’.

Limited outcome data were available for ‘gynecomastia’ and ‘cerebrovascular events’. As these are very rare events with considerable delayed onset in younger children, it’s likely that they are not well documented in these short-term randomised trials of only 63 days follow-up [11]. Therefore, we recommend that both antipsychotics are administered with caution to children and adolescents until more solid evidence about the risks of hormonal imbalances and infertility is available from trials with long term follow-up data.

The combination of IPD and the corresponding CSR are the most comprehensive and trustworthy source of Johnson & Johnson trial evidence available to date. However, a significant limitation of this study is that we were unable to access all company data and CSRs for 22 further eligible trials, which meant that this review was not entirely systematic and may be subject to selection bias. We made several requests for CSRs for Paliperidone trials at the European Medicines Agency, but one ongoing court case [38, 39] meant that they were unable to provide access to the CSR for seven further trials. Thus, to alleviate the potential for selection bias, we inspected the 22 studies and found that 15 of the studies did not appear to report any of the relevant adverse event’s data or provide the overall number of adverse events, and the other 7 studies did not provide amendable ‘arm-level’ data for inclusion in meta-analysis. Furthermore, the baseline scores for the primary outcome PANSS were measured using only one of the subscales in 13 of the studies and therefore the total PANSS score could not be estimated; the other 9 studies did not provide amenable PANSS data meaning they could not be meta-analysed with the IPD.

Another limitation of the study is that we did not compare the effects of all other efficacy outcomes using IPD and only a subset of AEs that were considered controversial as informed by earlier meta-analyses or previous ‘black box’ warnings were assessed using the patient safety listings data. Nevertheless, we did compare the PANSS total score using all available IPD provided, and CSRs conveniently supplemented where IPD for the outcome were unavailable. Moreover, comparing the differences of the effect-sizes between two different data sources by using the ratio of means/relative risk when there is clear overlap of studies and a lack of independence could well lead to unwieldly results. However, this was the only way to compare significance between the pooled effects of the data sources with any real confidence and precision.

The benefits of all three antipsychotics were more conservative than reported in the earlier studies restricted to only publicly available sources (journal publications and registry reports) [2, 10, 27, 28]. A less than 20% clinical improvement of Risperidone, Paliperidone and Paliperidone palmitate was observed whereas earlier studies showed between 20 and 50% clinical improvement.

The poor or miss reporting on harms in journal publications and registry reports was also concerning and disappointing, but it was in line with other findings looking at different pharmacological treatments [100, 101]. The pooled estimates from CSRs revealed a significant increase in risk across several AEs, which signifies the importance of CSRs and their use in supporting evidence-based practices. The thresholds for reporting harms across all sources of data could also be problematic, especially as rare events such as hyperprolactinaemia, gynecomastia and cerebrovascular events usually fall below the 5% or 2.5% incidence threshold which is often used in these populations and CSRs generally.

Platforms such as the YODA project and the Clinical Study Data Request [102] where the public can access IPD and CSRs from the manufacturers remain key and should be utilised more by systematic reviewers where possible. However, incorporating these data into systematic reviews requires better awareness of where these data can be sourced and greater resources [103–105]. In terms of data sourcing, regulatory agencies such as the European Medicines Agency [37] and Health Canada [106] have committed to releasing CSRs for trials on products that are centrally licenced. This will provide opportunities for investigators to freely access CSRs easily by ‘click and download’ for newly licenced interventional products. The Food and Drug Administration in the USA also launched its pilot scheme back in 2018, which is similar of that of the EMAs clinical trials data sharing policy. But access still remains largely restricted with few known cases of anyone accessing trial data/documents to date [107].

Moreover, in the UK, there exists a current policy drive towards improving transparency and open access of clinical trial data [108]. In terms of resource and time involvement, the challenge for funders of research grant applications is deciding where such comprehensive evidence syntheses are likely to make a difference and prioritising their funding even though they require greater resource and time demands. A framework approach would help support groups grappling with how to respond to the increasing availability of these new sources of information. It would likely even increase the scientific return on the funder's investment in the trial and most importantly the benefits to the public and future patients.

Calls for improved reporting standards, especially for harm outcomes in journal publications, continues to be debated even after the introduction of reporting guidelines such as the CONSORT harms [100, 109–111]. Many journals now allow for the publication of segments of de-identified IPD, redacted sections of CSRs including tables or narratives of harms alongside the main journal publication as supplementary online material. However, not all trials provide these levels of data, and they are still not as exhaustive as the full CSRs and/or IPD. The development of a central repository where all available redacted CSRs can be indexed and uploaded could help to alleviate some of the challenges associated with accessing and using these data. Such a development would require full co-operation and backing from all key stakeholders.

## Conclusions

Using IPD from the drug manufacturer, we found that Risperidone and Paliperidone lead to small clinical improvements in patients with schizophrenia or bipolar disorder which are more conservative compared to those of previous studies based on publicly available data. CSRs also contained a significantly greater volume of major harms compared to journal articles and trial registry reports. Our findings clearly support the use of IPD and CSRs for accurately assessing the efficacy and safety of antipsychotic drug interventions. For this type of in-depth analysis, more informed guidance is needed to help encourage researchers to access and analyse these data sources in an efficient manner.

## Supplementary Information

**Additional file 1.** Tables S1 – Medline, Embase, CENTRAL and PsycInfo searches.

**Additional file 2.** Table S2 Characteristics, content, and comparisons of reported data.

**Additional file 3.** Table S3 Characteristics of the participants in the trials and interventions involved and record of data available.

**Additional file 4.** Table S4 Additional information about the registry reports, clinical study reports and individual patient ‘listings’ data provided.

**Additional file 5.** Table S5 Content of information and details in the clinical study reports.

**Additional file 6.** Table S6 Risk of bias assessment utilising all sources of information.

**Additional file 7.** Table S7 Differential effects of Risperidone, Paliperidone and Paliperidone Palmitate separately in subgroups of the IPD meta-analysis based on primary outcome PANSS total score.

**Additional file 8.** Table S8 Meta-analysis of efficacy and harm outcomes across all three sources of data.

**Additional file 9.** Table S9 Relative risk and ratio of relative risk between document sources for total AEs and SAEs.

**Additional file 10.** Fig S1 Safety outcome reporting (AEs, SAEs and discontinuations) by study level.

**Additional file 11.** Table S10 Sensitivity analysis and narrative assessment of cause of death and gynecomastia cases.

**Additional file 12.** Table S11 Effect estimates based on condition (schizophrenia or bipolar disorder).

**Additional file 13.** Table S12 Effect estimates based on treatment dose for the three outcomes PANSS, AEs and SAEs.

**Additional file 14.** Fig S2 Forest plots of all meta-analysis.

**Additional file 15.** Fig S3 – Sensitivity analysis of low RoB studies

## Data Availability

As part of the broader YODA project, Johnson & Johnson have agreed to provide clinical research data on Risperidone, Paliperidone and Paliperidone palmitate to external investigators on request. Details of the data release policy are available from the YODA website at: https://yoda.yale.edu/sites/default/files/files/YODA%20Project%20Data%20Release%20Procedures%20February%202019.pdf
