# Supplementary material for: Benefits and harms of Risperidone and Paliperidone for treatment of patients with schizophrenia or bipolar disorder: a meta-analysis involving individual participant data and clinical study reports
Source: BMC Med. 2021 Aug 25;19:195. doi: 10.1186/s12916-021-02062-w (PMC8386072; doi:10.1186/s12916-021-02062-w)
Supplement: Supplementary file 1 — Additional file 1. Tables S1 – Medline, Embase, CENTRAL and PsycInfo searches. [file 12916_2021_2062_MOESM1_ESM.docx]

**Supplementary online material**

Contents

[Additional file 1: Table S1: Search strategy 3](#_Toc73625464)

[Additional file 2: Table S2: Characteristics, content, and comparison of reporting data 7](#_Toc73625465)

[Additional file 3: Table S3: Characteristics of the participants in the trials and interventions 8](#_Toc73625466)

[Additional file 4: Table S4: Additional information about information in report/data 14](#_Toc73625467)

[Additional file 5: Table S5: Content of information and details in the clinical study reports 17](#_Toc73625468)

[Additional file 6: Table S6: Risk of bias assessment utilising all sources of information 20](#_Toc73625469)

[Additional file 7: Table S7: Differential effects of treatment in subgroups of the IPD meta-analysis based on primary outcome PANSS total score 22](#_Toc73625470)

[Additional file 8: Table S8: Meta-analysis of efficacy/harm outcomes across all sources of data 24](#_Toc73625471)

[Additional file 9: Table S9: Relative risk and RRR between document sources for total AEs/SAEs 27](#_Toc73625472)

[Additional file 10: Fig S1: Safety outcome reporting by study level 28](#_Toc73625473)

[Additional file 11: Table S10: Sensitivity analysis and narrative assessments 31](#_Toc73625474)

[Additional file 12: Table S11: Effect estimates based on condition 33](#_Toc73625475)

[Additional file 13: Table S12: Effect estimates based on dose for PANSS, AEs and SAEs 34](#_Toc73625476)

[Additional file 14: Fig S2: Forest plot for all meta-analyses 35](#_Toc73625477)

[Additional file 15: Fig S3: Sensitivity analysis of Low RoB studies 124](#_Toc73625478)

# Additional file 1: Table S1: Search strategy

**Medline***

| **# ▲** | **Searches** | **Results** |
| --- | --- | --- |
| 1 | Risperidone.mp. or Risperidone/ | 7559 |
| 2 | Risperdal.mp. | 59 |
| 3 | RISPERDAL CONSTA.mp. | 15 |
| 4 | Paliperidone.mp. | 704 |
| 5 | Invega.mp. | 13 |
| 6 | Trevicta.mp. | 2 |
| 7 | Xeplion.mp. | 3 |
| 8 | or/1-7 | 7964 |
| 9 | exp Schizophrenia/ or schizophrenia.mp. | 135006 |
| 10 | exp Psychosis/ | 117770 |
| 11 | schizo$.mp. | 145628 |
| 12 | hebephreni$.mp. | 545 |
| 13 | oligophreni$.mp. | 523 |
| 14 | psychotic$.mp. | 60259 |
| 15 | psychosis.mp. | 56209 |
| 16 | psychoses.mp. | 15460 |
| 17 | bipolar disorder.mp. or Bipolar Disorder/ | 41125 |
| 18 | cyclothymic disorder.mp. or Cyclothymic Disorder/ | 606 |
| 19 | or/10-18 | 224191 |
| 20 | ((singl$ or doubl$ or trebl$ or tripl$) adj (blind$ or mask$)).mp. | 26620 |
| 21 | (random$ adj5 (assign$ or allocat$)).mp. | 45846 |
| 22 | randomi$.mp. | 93481 |
| 23 | crossover.mp. | 7714 |
| 24 | or/20-23 | 140915 |
| 25 | 8 and 19 and 24 | 1347 |
| **26** | **limit 26 to yr="2019 -Current"** | **51** |

**Embase***

| **# ▲** | **Searches** | **Results** |
| --- | --- | --- |
| 1 | Risperidone.mp. or Risperidone/ | 38111 |
| 2 | Risperdal.mp. | 2252 |
| 3 | RISPERDAL CONSTA.mp. | 266 |
| 4 | Paliperidone.mp. | 4821 |
| 5 | Invega.mp. | 366 |
| 6 | Trevicta.mp. | 11 |
| 7 | Xeplion.mp. | 52 |
| 8 | or/1-7 | 39745 |
| 9 | exp Schizophrenia/ or schizophrenia.mp. | 208887 |
| 10 | exp Psychosis/ | 284213 |
| 11 | schizo$.mp. | 235099 |
| 12 | hebephreni$.mp. | 929 |
| 13 | oligophreni$.mp. | 1291 |
| 14 | psychotic$.mp. | 56858 |
| 15 | psychosis.mp. | 129903 |
| 16 | psychoses.mp. | 10180 |
| 17 | bipolar disorder.mp. or Bipolar Disorder/ | 66317 |
| 18 | cyclothymic disorder.mp. or Cyclothymic Disorder/ | 955 |
| 19 | or/9-18 | 386531 |
| 20 | (clin$ adj2 trial).mp. | 1599490 |
| 21 | ((singl$ or doubl$ or trebl$ or tripl$) adj (blind$ or mask$)).mp. | 316386 |
| 22 | (random$ adj5 (assign$ or allocat$)).mp. | 201024 |
| 23 | randomi$.mp. | 1278435 |
| 24 | crossover.mp. | 102456 |
| 25 | exp randomized-controlled-trial/ | 636265 |
| 26 | exp crossover-procedure/ | 65556 |
| 27 | exp randomization/ | 89609 |
| 28 | or/20-27 | 2375892 |
| 29 | 8 and 19 and 28 | 8360 |
| **30** | **limit 30 to yr="2019 -Current"** | **361** |

**CENTRAL***

| **# ▲** | **Searches** | **Results** |
| --- | --- | --- |
| 1 | Risperidone.mp. or Risperidone/ | 3704 |
| 2 | Risperdal.mp. | 118 |
| 3 | RISPERDAL CONSTA.mp. | 41 |
| 4 | Paliperidone.mp. | 659 |
| 5 | Invega.mp. | 27 |
| 6 | Trevicta.mp. | 2 |
| 7 | Xeplion.mp. | 9 |
| 8 | or/1-7 | 4096 |
| 9 | exp Schizophrenia/ or schizophrenia.mp. | 17526 |
| 10 | exp Paranoid Disorders/ | 92 |
| 11 | schizo$.mp. | 18774 |
| 12 | hebephreni$.mp. | 38 |
| 13 | oligophreni$.mp. | 17 |
| 14 | psychotic$.mp. | 7216 |
| 15 | psychosis.mp. | 6636 |
| 16 | psychoses.mp. | 596 |
| 17 | bipolar disorder.mp. or Bipolar Disorder/ | 5746 |
| 18 | cyclothymic disorder.mp. or Cyclothymic Disorder/ | 28 |
| 19 | or/9-18 | 28542 |
| 20 | exp clinical trial/ | 163 |
| 21 | exp randomized controlled trials/ | 7991 |
| 22 | exp cross-over studies/ | 37577 |
| 23 | randomized controlled trial.pt. | 505478 |
| 24 | clinical trial.pt. | 279974 |
| 25 | controlled clinical trial.pt. | 91759 |
| 26 | (clinic$ adj2 trial).mp. | 433160 |
| 27 | (random$ adj5 control$ adj5 trial$).mp. | 629946 |
| 28 | (crossover or cross-over).mp. | 106538 |
| 39 | ((singl$ or double$ or trebl$ or tripl$) adj (blind$ or mask$)).mp. | 386791 |
| 30 | randomi$.mp. | 1014515 |
| 31 | (random$ adj5 (assign$ or allocat$ or assort$ or reciev$)).mp. | 218900 |
| 32 | or/20-31 | 1385069 |
| 33 | 8 and 19 and 32 | 2700 |
| **34** | **limit 34 to yr="2019 -Current"** | **144** |

**PsycInfo***

| **# ▲** | **Searches** | **Results** |
| --- | --- | --- |
| 1 | Risperidone.mp. or Risperidone/ | 7559 |
| 2 | Risperdal.mp. | 59 |
| 3 | RISPERDAL CONSTA.mp. | 15 |
| 4 | Paliperidone.mp. | 704 |
| 5 | Invega.mp. | 13 |
| 6 | Trevicta.mp. | 2 |
| 7 | Xeplion.mp. | 3 |
| 8 | or/1-7 | 7964 |
| 9 | exp Schizophrenia/ or schizophrenia.mp. | 135006 |
| 10 | exp Psychosis/ | 117770 |
| 11 | schizo$.mp. | 145628 |
| 12 | hebephreni$.mp. | 545 |
| 13 | oligophreni$.mp. | 523 |
| 14 | psychotic$.mp. | 60259 |
| 15 | psychosis.mp. | 56209 |
| 16 | psychoses.mp. | 15460 |
| 17 | bipolar disorder.mp. or Bipolar Disorder/ | 41125 |
| 18 | cyclothymic disorder.mp. or Cyclothymic Disorder/ | 606 |
| 19 | or/9-18 | 224191 |
| 20 | ((singl$ or doubl$ or trebl$ or tripl$) adj (blind$ or mask$)).mp. | 26620 |
| 21 | (random$ adj5 (assign$ or allocat$)).mp. | 45846 |
| 22 | randomi$.mp. | 93481 |
| 23 | crossover.mp. | 7714 |
| 24 | or/20-23 | 140915 |
| 25 | 8 and 19 and 24 | 1347 |
| **26** | **limit 26 to yr="2019 -Current"** | **51** |

*All searches were updated from 2019 onwards following Huhn et al, 2019.

Huhn M, Nikolakopoulou A, Schneider-Thoma J, et al. Comparative efficacy and tolerability of 32 oral antipsychotics for the acute treatment of adults with multi-episode schizophrenia: a systematic review and network meta-analysis. *The Lancet* 2019;394(10202):939-51. doi: 10.1016/S0140-6736(19)31135-3
