# Supplementary material for: Benefits and harms of Risperidone and Paliperidone for treatment of patients with schizophrenia or bipolar disorder: a meta-analysis involving individual participant data and clinical study reports
Source: BMC Med. 2021 Aug 25;19:195. doi: 10.1186/s12916-021-02062-w (PMC8386072; doi:10.1186/s12916-021-02062-w)
Supplement: Supplementary file 2 — Additional file 2. Table S2 Characteristics, content, and comparisons of reported data. [file 12916_2021_2062_MOESM2_ESM.docx]

# Additional file 2: Table S2: Characteristics, content, and comparison of reporting data

The following data on the characteristics, content and comparison of reporting were extracted.

| **Reporting criterion** | **Description of information extracted** |
| --- | --- |
| Characteristics of the trials | - Country - Sample size - Gender and age - Condition focus - Intervention and dose(s) used |
| Content of the different document types and availability of data | - Clinical study reports (CSRs): number of pages, redactions, availability of key sections of the reports (i.e. efficacy evaluation (E3 section 11*), primary and secondary outcome results, safety evaluation (E3 section 12*), tables of adverse events (AEs) and serious adverse events (SAEs), incidence-threshold of harms reported and patient safety narratives) - Individual patient data (IPD): demographic, efficacy, and AE listings - Registry reports: whether results were posted, date of when they were posted and last updated. |
| Comparison of reporting across CSRs, trial registries and journal publications of information based on | - Design aspects: randomisation, allocation, and blinding - Statistical analysis: sample size calculations - Reporting of efficacy outcomes: PANSS, time until relapse, CGI-S or YMRS - Reporting of harm outcomes: AEs/SAEs, discontinuation due to AE and death (with cause). |

*See <https://database.ich.org/sites/default/files/E3_Guideline.pdf>; PANSS: Positive and Negative Syndrome Scale; CGI-S: Clinical Global Impression rating scales; YMRS: Young Mania Rating Scale.
