# Supplementary material for: Benefits and harms of Risperidone and Paliperidone for treatment of patients with schizophrenia or bipolar disorder: a meta-analysis involving individual participant data and clinical study reports
Source: BMC Med. 2021 Aug 25;19:195. doi: 10.1186/s12916-021-02062-w (PMC8386072; doi:10.1186/s12916-021-02062-w)
Supplement: Supplementary file 3 — Additional file 3. Table S3 Characteristics of the participants in the trials and interventions involved and record of data available. [file 12916_2021_2062_MOESM3_ESM.docx]

# Additional file 3: Table S3: Characteristics of the participants in the trials and interventions involved and record of responses

**Characteristics of the participants in the trials and interventions involved**

| **Study ID** | **Country** | **Sample size** | **Condition** | **Intervention (delivery)** | **Length** | **Doses** | **Gender (% male)** | **Age (mean (sd) years)** | **Registry ID** | **Publication lead author (date published)** | **Trial register results posted** | **Clinical study report (full)** |
| --- | --- | --- | --- | --- | --- | --- | --- | --- | --- | --- | --- | --- |
| RIS-USA-72 | USA | 246 | Schizophrenia | Risperidone (oral) | 28 days | 4, 8 mg | 79% | Median: 38.5 (19-65) | NA | NA | NA | Yes^*^ |
| RIS-BIM-301 | USA | 169 | Bipolar disorder | Risperidone (oral) | 3 weeks | 0.5-2.5 or 3-6 mg | 49% | 13 (10-17) | NCT00076115 | Haas 2009 | No | Yes |
| RIS-SCH-302 | Greece | 160 | Schizophrenia | Risperidone (oral) | 6 weeks | 1-3 or 4-6 mg | 64% | 15.6 (1.3) | NCT00088075 | Haas 2009 | No | Yes |
| RIS-BIP-302 | USA | 275 | Bipolar disorder | Risperidone (intramuscular injection) | 52 weeks | 1-2 mg | 71% | 40 (11.8) | NCT00094926 | Macfadden 2009(1), 2009(2), 2011 | No | Yes |
| RISBIM3003 | Spain | 559 | Bipolar disorder | Risperidone (intramuscular injection) | 24 months | 25mg | 49% | 39 (11.8) | NCT00132678 | Quiroz 2010 | Yes | Yes |
| RIS-USA-121 | USA | 458 | Schizophrenia | Risperidone (intramuscular injection) | 12 weeks | 25, 50, 75 mg | > 65% | > 36.2 (9.5) | NCT00253136 | Kane 2003 | No | Yes |
| RIS-USA-102 | USA | 158 | Bipolar disorder | Risperidone (oral) | 3 weeks | 2, 4, 6, 12 mg | 50% | 41 (18-61) | NCT00253149 | Sachs 2002 | No | Yes |
| RIS-INT-69 | Russia | 439 | Bipolar disorder | Risperidone (intramuscular injection) | 12 weeks | 1-6 mg | 53.9 | 41.3 (13.1) | NCT00253162 | Smulevich 2005 | No | Yes |
| RIS-USA-239 | USA | 267 | Bipolar disorder | Risperidone (oral) | 3 weeks | 1-6 mg | 53% | 38.1 (11.9) | NCT00257075 | Hirschfeld 2004 | No | Yes |
| RISBMN3001 | Spain | 585 | Bipolar disorder | Risperidone (intramuscular injection) | 18 months | 25, 50mg | 48% | 36.9 (11.4) | NCT00391222 | Vieta 2012 | Yes | Yes |
| RIS-SCP-402 | USA | 381 | Schizophrenia | Risperidone (oral) | 6 weeks | 4, 6 mg | 69% | 34.7 (9.6) | NCT00061802 | Gharabawi 2006 | No | Yes |
| R076477-SCH-304 | USA | 444 | Schizophrenia | Paliperidone (oral) | 6 weeks | 6, 12 mg | 74% | 41.6 (10.67) | NCT00077714 | Marder 2007 | No | Yes |
| R076477-SCH-303 | USA | 628 | Schizophrenia | Paliperidone (oral) | 6 weeks | 6, 9, 12 mg | 52% | 37.1 (10.9) | NCT00078039 | Kane 2007 | No | Yes |
| R076477-SCH-302 | Greece | 114 | Schizophrenia | Paliperidone (oral) | 24 weeks | 3-12 mgs | 27% | 70 | NCT00085748 | Tzimos 2008 | No | Yes |
| R076477-SCH-301 | USA | 111 | Schizophrenia | Paliperidone (oral) | 14 weeks | 3-15 mgs | 56% | 39 (10.7) | NCT00086320 | Kramer 2007 | No | Yes |
| R076477-SCH-1010 | France | 36 | Schizophrenia | Paliperidone (oral) | 14 days | 9 mg | 67% | 32.2 (7.32) | NCT00105326 | Luthringer 2007 | No | Yes |
| R076477-BIM-3001 | USA | 469 | Acute mania | Paliperidone (oral) | 3 weeks | 3, 6, or 12mgs | 55% | 39 (11) | NCT00299715 | Berwaerts 2012 | No | Yes |
| R076477-BIM-3003 | USA | 300 | Bipolar 1 disorder | Paliperidone (oral) | 6 weeks | 3-12 mg | 57% | 40 (10.9) | NCT00309686 | Berwaerts 2011 | No | Yes |
| R076477-BIM-3002 | Spain | 493 | Bipolar 1 disorder | Paliperidone (oral) | 12 weeks | 3-12 mg | 58% | 40 (11.3) | NCT00309699 | Vieta 2010 | No | Yes |
| R076477SCH3015 | USA | 399 | Schizophrenia | Paliperidone (oral) | 6 weeks | 9, 12 mgs | 70% | 35.7 (11.6) | NCT00334126 | Canuso 2009 | No | Yes |
| R076477SCA3001 | USA | 316 | Schizophrenia | Paliperidone (oral) | 6 weeks | 6-12 mgs | 66.70% | 38.1 (10) | NCT00397033 | Canuso 2010 | Yes | Yes |
| R076477SCA3002 | USA | 307 | Schizophrenia | Paliperidone (oral) | 6 weeks | 3-12 mg | 67.00% | 31.2 (9.9) | NCT00412373 | Canuso 2010 | Yes | Yes |
| R076477-SCH-701 | USA | 235 | Schizophrenia | Paliperidone (oral) | 52 weeks | Flexible: 3-15mg; 9 mg starting | 66% | 35.8 (9.83) | NCT00645307 | Kramer 2010 | No | Yes |
| R076477-SCH-702 | Greece | 114 | Schizophrenia | Paliperidone (oral) | 6 weeks | 3-12 mgs | 27% | 70 | NCT00752427 | Tzimos 2008 | No | Yes |
| R092670-SCH-201 | USA | 250 | Schizophrenia | Paliperidone palmitate (intramuscular injection) | 9 weeks | 50, 100 mg eq. | 62% | 40 (10.5) | NCT00074477 | Kramer 2010 | No | Yes |
| R092670PSY3004 | USA | 518 | Schizophrenia | Paliperidone palmitate (intramuscular injection) | 13 weeks | 25, 50, 100 mg eq | 67% | 40.8 (11.3) | NCT00101634 | Nasrallah 2010 | No | Yes |
| R092670PSY3001 | USA | 414 | Schizophrenia | Paliperidone palmitate (intramuscular injection) | 24 weeks | 25, 50, 100 mg eq | 55% | 40 (11.5) | NCT00111189 | Hough 2010 | No | Yes |
| R092670PSY3003 | USA | 366 | Schizophrenia | Paliperidone palmitate (intramuscular injection) | 13 weeks | 25, 100, 150 mg eq | 69% | 40 (10.8) | NCT00210548 | Gopal 2010 | No | Yes |
| R092670PSY3007 | USA | 652 | Schizophrenia | Paliperidone palmitate (intramuscular injection) | 13 weeks | 25, 100, 150 mg eq | 67% | 39 | NCT00590577 | Pandina 2010 | Yes | Yes |
| R092670SCA3004 | USA | 667 | Schizophrenia | Paliperidone palmitate (intramuscular injection) | 15 months | Flexable: 25 - 100 mg | 51% | 38.6 (19-66) | NCT01193153 | Fu 2015 | Yes | Yes |
| PALM-JPN-4 | Japan | 323 | Schizophrenia | Paliperidone palmitate (intramuscular injection) | 13 weeks | 75, 100, 150 mg ep. | 57% | 44.8 (12.99) | NCT01299389 | Akihide 2013 | Yes | Yes |
| R092670PSY3012 | USA | 509 | Schizophrenia | Paliperidone palmitate (intramuscular injection) | 12 weeks | 50, 75, 100 or 150 mg eq | 75% | 37.8 (11.01) | NCT01529515 | Berwaerts 2015 | Yes | Yes |
| R076477PSZ3001 | USA | 201 | Schizophrenia | Paliperidone (oral) | 6 weeks | 1.5, 3, 6 mgs | 59% | 15.4 (1.53) | NCT00518323 | Singh 2011 | Yes | Yes |
| R076477-SCH-3041 | China | 135 | Schizophrenia | Paliperidone (oral) | 24 weeks | 3-12 mg | 41% | 31.7 (10.90) | NCT01662310 | Rui 2014 | Yes | Yes |
| R076477-SCH-305 | USA (mostly) | 618 | Schizophrenia | Paliperidone (oral) | 6 weeks | 3,9,15 mgs | 68% | 36.8 (10.6) | NCT00083668 | Davidson 2007 | No | Yes |

*Not full report but main content of the core report was available for meta-analysis; NA: not applicable; SD: standard deviation.

**Record of responses from data custodians and regulators following requests for data**

| **Data custodian** | **Response** |
| --- | --- |
| YODA | Dear Investigator,  Your inquiry about data availability (**NCT01086748, NCT00694707, and NCT00272584**) has been reviewed by the YODA Project. The Data Partner has confirmed that these data cannot be made available to external investigators for the following reason:  1. The Data Partner is not the clinical trial sponsor.  Please refer to the YODA Project Policy and Procedures documentation for a more thorough explanation of the circumstances which may result in a Data Partner being unable to make clinical research data available to external investigators. Please note that Johnson & Johnson and SI-BONE are the only Data Partners currently making clinical trial data available through the YODA Project. As such, the YODA Project will not be able to provide data owned by other clinical trial sponsors. Please verify the trial sponsor at www.ClinicalTrials.gov prior to submitting an inquiry.  We encourage you to regularly check the list of currently available data (available here) in case new data become available that are relevant to your research.  Thank you again for your inquiry and interest in this project. If you have any questions at any point, please contact the YODA Project at yodap@yale.edu.  Best regards, The YODA Project |
| YODA | Dear Investigator,  Your inquiry about data availability (**NCT00061802**) has been reviewed by the YODA Project. The Data Partner has confirmed that this study can be made available for sharing. Please note that only clinical documents are available – participant-level data is not available for this study. The Trial List has been updated to reflect this newly available study.   If you are still interested in these data, please submit a formal research request on the YODA Project Data Request Website. More information about what is required to submit a request can be found here.  Thank you again for your inquiry and interest in this project. If you have any questions at any point, please contact the YODA Project at yodap@yale.edu.  Best regards, The YODA Project |
| YODA | Dear Investigator,  The remaining studies from your inquiry have been reviewed by the YODA Project:  • PMID: 18545059 – This is a post hoc analyses of pooled studies • PMID: 19552963 – This is a post hoc analyses of pooled studies • PMID: 19560322 – This is a post hoc analyses of pooled studies • PMID: 20199482 – This is a post hoc analyses of pooled studies  Thank you again for your inquiry and interest in this project. If you have any questions at any point, please email us at yodap@yale.edu.  Best regards, The YODA Project |
| EMA | Response had to be removed as it contained personal confidential information. But two CSRs were retrieved from the European Medicines Agency Clinical Data online portal: https://clinicaldata.ema.europa.eu/web/cdp/home |
| Vivli | Revealed the same studies listed at the YODA project |
| Health Canada | No trials were identified |
| FDA | Drug approval packages were identified for Risperdal, but no further trials or data were identified |
