# Supplementary material for: Benefits and harms of Risperidone and Paliperidone for treatment of patients with schizophrenia or bipolar disorder: a meta-analysis involving individual participant data and clinical study reports
Source: BMC Med. 2021 Aug 25;19:195. doi: 10.1186/s12916-021-02062-w (PMC8386072; doi:10.1186/s12916-021-02062-w)
Supplement: Supplementary file 4 — Additional file 4. Table S4 Additional information about the registry reports, clinical study reports and individual patient ‘listings’ data provided. [file 12916_2021_2062_MOESM4_ESM.docx]

# Additional file 4: Table S4: Additional information about registry reports, clinical study reports and individual patient ‘listings’ data provided

|  | **Registry Reports** | | | **Clinical study reports** | | | | **Individual patient listings** | | |
| --- | --- | --- | --- | --- | --- | --- | --- | --- | --- | --- |
| **Study ID** | **Trial register results posted** | **Date posted (results posted)** | **Last updated** | **Clinical study report (full)** | **Annotated Case Report Form** | **Complete Case report forms** | **Statistical analysis plan** | **Demographic ^φ^** | **Efficacy ^φ^** | **Safety ^φ^** |
| RIS-USA-72 | NA | NA | NA | No | No | No | No | Yes | Yes | Yes |
| RIS-BIM-301 | No | Jan-04 | Jun-11 | Yes | Yes | No | Yes | Yes | Yes | Yes |
| RIS-SCH-302 | No | Jul-04 | Jun-11 | Yes | Yes | No | Yes | Yes | Yes | Yes |
| RIS-BIP-302 | No | Oct-04 | May-11 | Yes | No | No | Yes | Yes | Yes | Yes |
| RISBIM3003 | Yes | August 2005 (February 2009) | Jul-13 | Yes | Yes | Not provided, but they do mention in report that they do provide in appendix of CSR for deaths and other SAEs, withdrawals | Yes | Yes | Yes | Yes |
| RIS-USA-121 | No | Nov-05 | Dec-10 | Yes | Yes | No | Yes | Yes | Yes | Yes |
| RIS-USA-102 | No | Nov-05 | Jan-11 | Yes | Yes | No | Yes | Yes | Yes | Yes |
| RIS-INT-69 | No | Nov-05 | Jan-11 | Yes | Yes | No | Yes | Yes | Yes | Yes |
| RIS-USA-239 | No | Nov-05 | Jan-11 | Yes | Yes | No | Yes | Yes | Yes | Yes |
| RISBMN3001 | Yes | October 2006 (November 2010) | May-14 | Yes | Yes | No | Yes | Yes | Yes | Yes |
| RIS-SCP-402 | No | Jun-03 | Feb-12 | Yes | No | No | Yes | No | No | No |
| R076477-SCH-304 | No | Feb-04 | Jun-11 | Yes | Yes | No | Yes | Yes | Yes | Yes |
| R076477-SCH-303 | No | Mar-04 | Jun-11 | Yes | Yes | No | Yes | Yes | Yes | Yes |
| R076477-SCH-302 | No | Jun-04 | Jun-11 | Yes | Yes | No | Yes | Yes | Yes | Yes |
| R076477-SCH-301 | No | Jul-04 | Jun-11 | Yes | Yes | No | Yes | Yes | Yes | Yes |
| R076477-SCH-1010 | No | Mar-05 | May-10 | Yes | Yes | No | No | Yes | Yes | Yes |
| R076477-BIM-3001 | No | Mar-06 | Jun-11 | Yes | Yes | Not provided, but does mention in CSR some detailed information for certain subjects | Yes | Yes | Yes | Yes |
| R076477-BIM-3003 | No | Apr-06 | May-11 | Yes | Yes | Not provided, but does mention in CSR that representative written information for subject | Yes | Yes | Yes | Yes |
| R076477-BIM-3002 | No | Apr-06 | Jun-14 | Yes | Yes | Not provided, but does mention in CSR that representative written information for subject | Yes | Yes | Yes | Yes |
| R076477SCH3015 | No | Jun-06 | May-11 | Yes | Yes | No | Yes | Yes | Yes | Yes |
| R076477SCA3001 | Yes | November 2006 (July 2009) | Jul-13 | Yes | Yes | No | Yes | Yes | Yes | Yes |
| R076477SCA3002 | Yes | December 2006 (December 2009) | May-14 | Yes | Yes | No | Yes | Yes | Yes | Yes |
| R076477-SCH-701 | No | Mar-08 | Jun-11 | Yes | Yes | No | Yes | Yes | Yes | Yes |
| R076477-SCH-702 | No | Sep-08 | Jun-11 | Yes | Yes | No | Yes | Yes | Yes | Yes |
| R092670-SCH-201 | No | Dec-03 | May-11 | Yes | Yes | No | Yes | Yes | Yes | Yes |
| R092670PSY3004 | No | Jan-05 | Jun-11 | Yes | Yes | No | Yes | Yes | Yes | Yes |
| R092670PSY3001 | No | May-05 | Jun-14 | Yes | Yes | No | Yes | Yes | Yes | Yes |
| R092670PSY3003 | No | Sep-05 | Jun-11 | Yes | Yes | No | Yes | Yes | Yes | Yes |
| R092670PSY3007 | Yes | January 2008 (October 2009) | Jun-14 | Yes | Yes | No | Yes | Yes | Yes | Yes |
| R092670SCA3004 | Yes | September 2010 (January 2015) | Jan-15 | Yes | Yes | No | Yes | Yes | Yes | Yes |
| PALM-JPN-4 | Yes | February 2011 (June 2013) | Jun-13 | Yes | Yes | No | No | Yes | Yes | Yes |
| R092670PSY3012 | Yes | February 2012 (June 2015) | May-16 | Yes | Yes | No | Yes | Yes | Yes | Yes |
| R076477PSZ3001 | Yes | August 2007 (June 2010) | Apr-14 | Yes | Yes | No | Yes | Yes | Yes | Yes |
| R076477-SCH-3041 | Yes | August 2012 (June 2014) | Sep-14 | Yes | Yes | No | Yes | Yes | Yes | Yes |
| R076477-SCH-305 | No | May-04 | Jun-11 | Yes | Yes | No | Yes | Yes | Yes | Yes |

NA: not applicable; SAE: serious adverse events; CSR: clinical study reports.

^φ^ Full manufacturers data includes specific data for patient demographic data, adverse events dataset and datasets for primary efficacy outcomes such as PANSS and relapse etc.
