# Supplementary material for: Benefits and harms of Risperidone and Paliperidone for treatment of patients with schizophrenia or bipolar disorder: a meta-analysis involving individual participant data and clinical study reports
Source: BMC Med. 2021 Aug 25;19:195. doi: 10.1186/s12916-021-02062-w (PMC8386072; doi:10.1186/s12916-021-02062-w)
Supplement: Supplementary file 5 — Additional file 5. Table S5 Content of information and details in the clinical study reports. [file 12916_2021_2062_MOESM5_ESM.docx]

# Additional file 5: Table S5: Content of information and details in the clinical study reports

| **Study ID** | **Pages** | **Redactions*** | **Efficacy evaluation (E3 section 11)** | **Table of Primary & secondary results** | **Safety evaluation provided (E3 section 12)** | **Integrated summary of safety/conclusions** | **Table of AEs/SAEs** | **Incidence threshold used in any group** | **Narratives for serious adverse events or deaths** |
| --- | --- | --- | --- | --- | --- | --- | --- | --- | --- |
| RIS-USA-72 | 174 | High | Yes | Yes | Yes | No | No | At least 5% | Removed from CSR appendix |
| RIS-BIM-301 | 1186 | Medium | Yes | Yes | Yes | Yes | Yes | At least 5% | Removed from CSR appendix |
| RIS-SCH-302 | 632 | Low | Yes | Yes | Yes | Yes | Yes | At least 5% | Yes (some information retracted) |
| RIS-BIP-302 | 3859 | Medium | Yes | Yes | Yes | Yes | Yes | At least 2% | Removed from CSR appendix |
| RISBIM3003 | 1246 | Medium | Yes | Yes | Yes | Yes | Yes | At least 2% | Removed from CSR appendix |
| RIS-USA-121 | 989 | Medium | Yes | Yes | Yes | Yes | Yes | At least 5% | Removed from CSR appendix |
| RIS-USA-102 | 555 | Medium | Yes | Yes | Yes | Yes | Yes | At least 5% | Removed from CSR appendix |
| RIS-INT-69 | 1141 | Medium | Yes | Yes | Yes | Yes | Yes | At least 5% | Removed from CSR appendix |
| RIS-USA-239 | 389 | Medium | Yes | Yes | Yes | Yes | Yes | At least 5% | Removed from CSR appendix |
| RISBMN3001 | 1457 | Medium | Yes | Yes | Yes | Yes | Yes | At least 2% | Removed from CSR appendix |
| RIS-SCP-402 | 198 | Medium | Yes | Yes | Yes | Yes | Yes | At least 5% | Removed from CSR appendix |
| R076477-SCH-304 | 700 | Medium | Yes | Yes | Yes | Yes | Yes | At least 5% | Removed from CSR appendix |
| R076477-SCH-303 | 794 | Medium | Yes | Yes | Yes | Yes | Yes | At least 5% | Removed from CSR appendix |
| R076477-SCH-302 | 599 | Medium | Yes | Yes | Yes | Yes | Yes | At least 5% | Removed from CSR appendix |
| R076477-SCH-301 | 982 | Low | Yes | Yes | Yes | Yes | Yes | At least 5% | Yes (some information retracted) |
| R076477-SCH-1010 | 431 | Medium | Yes | Yes | Yes | Yes | Yes | At least 5% | Removed from CSR appendix |
| R076477-BIM-3001 | 554 | Medium | Yes | Yes | Yes | Yes | Yes | At least 5% | Removed from CSR appendix |
| R076477-BIM-3003 | 473 | Medium | Yes | Yes | Yes | Yes | Yes | At least 5% | Removed from CSR appendix |
| R076477-BIM-3002 | 797 | Medium | Yes | Yes | Yes | Yes | Yes | At least 5% | Removed from CSR appendix |
| R076477SCH3015 | 1087 | High | Yes | Yes | Yes | Yes | Yes | At least 5% | No mention |
| R076477SCA3001 | 2084 | Medium | Yes | Yes | Yes | Yes | Yes | At least 5% | Removed from CSR appendix |
| R076477SCA3002 | 1775 | Medium | Yes | Yes | Yes | Yes | Yes | At least 5% | Removed from CSR appendix |
| R076477-SCH-701 | 1813 | Medium | Yes | Yes | Yes | Yes | Yes | At least 5% | Removed from CSR appendix |
| R076477-SCH-702 | 842 | Low | Yes | Yes | Yes | Yes | Yes | At least 5% | Yes |
| R092670-SCH-201 | 771 | Medium | Yes | Yes | Yes | Yes | Yes | At least 2% | Removed from CSR appendix |
| R092670PSY3004 | 714 | Medium | Yes | Yes | Yes | Yes | Yes | At least 2% | Removed from CSR appendix |
| R092670PSY3001 | 949 | Medium | Yes | Yes | Yes | Yes | Yes | At least 2% | Removed from CSR appendix |
| R092670PSY3003 | 844 | High | Yes | Yes | Yes | Yes | Yes | At least 2% | Removed from CSR appendix |
| R092670PSY3007 | 666 | Medium | Yes | Yes | Yes | Yes | Yes | At least 2% | Removed from CSR appendix |
| R092670SCA3004 | 1338 | Medium | Yes | Yes | Yes | Yes | Yes | At least 2% | Removed from CSR appendix |
| PALM-JPN-4 | 668 | Medium | Yes | Yes | Yes | Yes | Yes | At least 2% | Removed from CSR appendix |
| R092670PSY3012 | 1296 | Medium | Yes | Yes | Yes | Yes | Yes | At least 2% | Removed from CSR appendix |
| R076477PSZ3001 | 825 | Medium | Yes | Yes | Yes | Yes | Yes | At least 5% | Removed from CSR appendix |
| R076477-SCH-3041 | 1066 | Medium | Yes | Yes | Yes | Yes | Yes | At least 2% | Removed from CSR appendix |
| R076477-SCH-305 | 182 | Medium | Yes | Yes | Yes | Yes | Yes | At least 5% | Removed from CSR appendix |

*Coding defined as: Low (i.e. only subject ID and PI names redacted); Moderate (i.e. identification of narratives and some pages being removed); High (i.e. pages were removed in the core report and/or some outcome data appears to have been redacted).

CSR: clinical study reports; AEs: adverse events; SAEs: Serious adverse events
