# Supplementary material for: Benefits and harms of Risperidone and Paliperidone for treatment of patients with schizophrenia or bipolar disorder: a meta-analysis involving individual participant data and clinical study reports
Source: BMC Med. 2021 Aug 25;19:195. doi: 10.1186/s12916-021-02062-w (PMC8386072; doi:10.1186/s12916-021-02062-w)
Supplement: Supplementary file 6 — Additional file 6. Table S6 Risk of bias assessment utilising all sources of information. [file 12916_2021_2062_MOESM6_ESM.docx]

# Additional file 6: Table S6: Risk of bias assessment utilising all sources of information

|  | **Selection bias** | | **Performance bias** | **Detection bias** | **Attrition bias** | **Reporting bias** |  | **Overall RoB** |
| --- | --- | --- | --- | --- | --- | --- | --- | --- |
| **Study ID** | *Random sequence generation* | *Allocation concealment* | *Blinding of participants and personnel* | *Blinding of outcome assessment* | *Incomplete outcome data* | *Selective reporting* | *Other bias* |  |
| RIS-USA-72 | Unclear | Unclear | Unclear | Unclear | Low* | Low* | High | Moderate |
| RIS-BIM-301 | Low | Low* | Low | Low* | Low | Low | Low | Low |
| RIS-SCH-302 | High | Low* | High | High | Low* | Low | Low* | High |
| RIS-BIP-302 | Unclear | Unclear | High | Low* | Low | Low | Low* | Moderate |
| RISBIM3003 | Low | Unclear | Low | Unclear | Low | Low | Low | Moderate |
| RIS-USA-121 | Low | Low | Low* | Low | Low* | Low* | Unclear | Low |
| RIS-USA-102 | Low | Low* | Low | Low | Low | Low* | High | Low |
| RIS-INT-69 | Low | Low | Unclear | Unclear | Low* | Low* | Low | Low |
| RIS-USA-239 | Unclear | Low | Low | Unclear | Low | Low | Low* | Low |
| RISBMN3001 | Low | Unclear | Low | Low | Low | Low* | Low | Low |
| RIS-SCP-402 | Low | Unclear | Unclear | Unclear | Unclear | Low* | High | Moderate |
| R076477-SCH-304 | Low | Low | Low* | Low* | Low | Low | Low | Low |
| R076477-SCH-303 | Low | Low | Low | Low | Low | Low* | Low* | Low |
| R076477-SCH-302 | Low* | Low* | Low | Low | Low* | Low* | High | Low |
| R076477-SCH-301 | Low* | Unclear | Low | Low* | Low* | Low* | High | Low |
| R076477-SCH-1010 | Low* | Low | Low* | Low* | High | Low* | High | Moderate |
| R076477-BIM-3001 | Low | Low* | Low | Low | Low | Low* | High | Moderate |
| R076477-BIM-3003 | Low | Low | Low | Low* | Low | Low* | High | Moderate |
| R076477-BIM-3002 | Low | Low* | Low | Low | Low | Low* | Low* | Low |
| R076477SCH3015 | Low* | Low | Low* | Low* | Unclear | Low | High | Moderate |
| R076477SCA3001 | Low | Low | Low | Low | High | Low* | Low* | Moderate |
| R076477SCA3002 | Low | Low | Low* | Low* | Low | Low* | Low* | Low |
| R076477-SCH-701 | Unclear | Unclear | High | High | Low | Low* | Low* | High |
| R076477-SCH-702 | Unclear | Unclear | High* | High* | Unclear | Low | Low | High |
| R092670-SCH-201 | Low | Low | Low | Low | Low* | Low* | Low | Low |
| R092670PSY3004 | Low* | Unclear | Low | Low* | Low | Low* | Low* | Low |
| R092670PSY3001 | Low | Low* | Low | Low | Low | Low* | Low | Low |
| R092670PSY3003 | Unclear | Low | Low* | Low* | Low* | Low | Low | Low |
| R092670PSY3007 | Low* | Low* | Low | Low | Low | Low* | Low* | Low |
| R092670SCA3004 | Low | Low | Low | Low* | Unclear | Low | Low* | Low |
| PALM-JPN-4 | Low | Low* | Low | Low* | High | Low | Low* | Moderate |
| R092670PSY3012 | Low* | Unclear | Low* | Low* | Low | Low | Low | Low |
| R076477PSZ3001 | Low | Low* | Low* | Low | Unclear | Low* | Low | Low |
| R076477-SCH-3041 | Low | Low | Low | Low* | Low | Low* | Low* | Low |
| R076477-SCH-305 | Low* | Low* | Low | Low | Low* | Low | Low* | Low |

*Judgement was downgraded after assessing the corresponding clinical study report
