# Supplementary material for: Benefits and harms of Risperidone and Paliperidone for treatment of patients with schizophrenia or bipolar disorder: a meta-analysis involving individual participant data and clinical study reports
Source: BMC Med. 2021 Aug 25;19:195. doi: 10.1186/s12916-021-02062-w (PMC8386072; doi:10.1186/s12916-021-02062-w)
Supplement: Supplementary file 7 — Additional file 7. Table S7 Differential effects of Risperidone, Paliperidone and Paliperidone Palmitate separately in subgroups of the IPD meta-analysis based on primary outcome PANSS total score. [file 12916_2021_2062_MOESM7_ESM.docx]

# Additional file 7: Table S7: Differential effects of risperidone, paliperidone and paliperidone palmitate separately in subgroups of the IPD meta-analysis based on primary outcome PANSS total score

|  |  |  | **Treatment covariate interaction** | | |
| --- | --- | --- | --- | --- | --- |
| **Characteristic** | **No. of studies (no. of participants)** | **Standardised mean difference* (95% CI)** | **Coefficient; 95% CI** | **P-value** | **I^2^ (95% CI) (%)** |
| **Risperidone:** |  |  |  |  |  |
| ¥Median Age: |  |  |  |  |  |
| - ≥ 30 years | 3 (458) | -0.319 (-0.656, 0.018) | 1 | NA | 11.58 (1.61, 51.09) |
| - < 30 years | 4 (673) | -0.283 (-0.603, 0.037) | 0.035 (-0.228, 0.299) | 0.792 |  |
| Age class: |  |  |  |  |  |
| - Under 18 | 4 (330) | -0.335 (-0.678, 0.007) | 1 | NA | 9.23 (0.75, 57.63) |
| - Young adult (18 to 29) | 3 (370) | -0.270 (-0.588, 0.048) | 0.065 (-0.250, 0.381) | 0.685 |  |
| - Adult (30 to 60) | 3 (413) | -0.313 (-0.632, 0.006) | 0.022 (-0.336, 0.381) | 0.903 |  |
| - Older adult (≥ 60) | 2 (18) | 0.162 (-0.645, 0.970) | 0.498 (-0.329, 1.324) | 0.238 |  |
| Gender: |  |  |  |  |  |
| - Male | 4 (775) | **-0.304 (-0.590, -0.018)** | 1 | NA | 9.85 (1.56, 42.93) |
| - Female | 4 (356) | -0.292 (-0.607, 0.024) | 0.013 (-0.203, 0.228) | 0.908 |  |
| Ethnicity: |  |  |  |  |  |
| - White | 4 (614) | -0.256 (-0.562, 0.050) | 1 | NA | 10.95 (1.60, 48.21) |
| - Black | 4 (328) | -0.334 (-0.677, 0.010) | -0.078 (-0.319, 0.164) | 0.529 |  |
| - Asian | 2 (59) | -0.266 (-0.816, 0.285) | -0.009 (-0.524, 0.505) | 0.972 |  |
| - Other | 4 (130) | **-0.518 (-0.928, -0.108)** | -0.262 (-0.587, 0.063) | 0.114 |  |
| **Paliperidone:** |  |  |  |  |  |
| ¥Median Age: |  |  |  |  |  |
| - ≥ 32 years | 11 (2213) | **-0.237 (-0.360, -0.113)** | 1 | NA | 3.74 (1.18, 11.25) |
| - < 32 years | 12 (1608) | **-0.364 (-0.501, -0.226)** | -0.127 (-0.261, 0.007) | 0.064 |  |
| Age class: |  |  |  |  |  |
| - Under 18 | 5 (217) | -0.056 (-0.467, 0.354) | 1 | NA | 4.88 (1.55, 14.28) |
| - Young adult (18 to 29) | 11 (959) | **-0.413 (-0.577, -0.250)** | -0.357 (-0.792, 0.781) | 0.108 |  |
| - Adult (30 to 60) | 11 (2469) | **-0.261 (-0.397, -0.126)** | -0.205 (-0.630, 0.220) | 0.345 |  |
| - Older adult (≥ 60) | 10 (176) | -0.302 (-0.637, 0.034) | -0.245 (-0.772, 0.281) | 0.361 |  |
| Gender: |  |  |  |  |  |
| - Male | 13 (2248) | **-0.262 (-0.390, -0.134)** | 1 | NA | 4.33 (1.47, 12.05) |
| - Female | 13 (1573) | **-0.320 (-0.457, -0.183)** | -0.058 (-0.177, 0.062) |  |  |
| Ethnicity: |  |  |  |  |  |
| - White | 13 (2285) | **-0.297 (-0.436, -0.157)** | 1 | NA | 5.72 (2.03, 15.05) |
| - Black | 11 (743) | **-0.276 (-0.459, -0.093)** | 0.021 (-0.140, 0.181) | 0.800 |  |
| - Asian | 9 (597) | **-0.368 (-0.565, -0.170)** | -0.071 (-0.249, 0.107) | 0.435 |  |
| - Other | 11 (196) | 0.116 (-0.177, 0.409) | **0.413 (0.137, 0.689)** | **0.003** |  |
| **Paliperidone Palmitate:** |  |  |  |  |  |
| ¥Median Age: |  |  |  |  |  |
| - ≥ 12 years | 2 (655) | **-0.496 (-0.695, -0.296)** | 1 | NA | 1.34 (0.06, 24.73) |
| - < 12 years | 3 (1554) | **-0.252 (-0.407, -0.097)** | 0.244 (-0.009, 0.496) | 0.058 |  |
| Age class: |  |  |  |  |  |
| - Under 18 | 2 (4) | -1.154 (-2.883, 0.576) | 1 | NA | 2.85 (0.34, 20.24) |
| - Young adult (18 to 29) | 5 (530) | **-0.463 (-0.675, -0.251)** | 0.690 (-1.041, 2.422) | 0.435 |  |
| - Adult (30 to 60) | 5 (1627) | **-0.287 (-0.449, -0.124)** | 0.867 (-0.861, 2.594) | 0.325 |  |
| - Older adult (≥ 60) | 5 (48) | **-0.996 (-1.571, -0.421)** | 0.157 (-1.657, 1.972) | 0.865 |  |
| Gender: |  |  |  |  |  |
| - Male | 5 (1438) | **-0.324 (-0.488, -0.160)** | 1 | NA | 2.71 (0.31, 20.0) |
| - Female | 5 (771) | **-0.381 (-0.568, -0.195)** | -0.057 (-0.223, 0.109) | 0.502 |  |
| Ethnicity: |  |  |  |  |  |
| - White | 5 (1285) | **-0.390 (-0.545, -0.236)** | 1 | NA | 2.05 (0.17, 20.55) |
| - Black | 5(624) | **-0.222 (-0.413, -0.031)** | 0.168 (-0.019, 0.355) | 0.078 |  |
| - Asian | 4 (255) | **-0.379 (-0.640, -0.119)** | 0.011 (-0.246, 0.269) | 0.933 |  |
| - Other | 5 (45) | -0.432 (-1.025, 0.161) | -0.041 (-0.632, 0.549) | 0.891 |  |

*Model accounted for baseline PANSS scores; ¥ Per year of age; yrs: years.
