# Supplementary material for: Benefits and harms of Risperidone and Paliperidone for treatment of patients with schizophrenia or bipolar disorder: a meta-analysis involving individual participant data and clinical study reports
Source: BMC Med. 2021 Aug 25;19:195. doi: 10.1186/s12916-021-02062-w (PMC8386072; doi:10.1186/s12916-021-02062-w)
Supplement: Supplementary file 8 — Additional file 8. Table S8 Meta-analysis of efficacy and harm outcomes across all three sources of data. [file 12916_2021_2062_MOESM8_ESM.docx]

# Additional file 8: Table S8: Meta-analysis of efficacy and harm outcomes across all three sources of data

|  | **Clinical study reports** | | | | | **Journal publication** | | | | | **Registry report** | | | | |
| --- | --- | --- | --- | --- | --- | --- | --- | --- | --- | --- | --- | --- | --- | --- | --- |
| **Outcomes** | **No. of studies** | **No. of patients on Intervention** | **No. of patients on Placebo** | **Effect estimate (95% CI)** | **I2 (95% CI), %** | **No. of studies** | **No. of patients on Intervention** | **No. of patients on Placebo** | **Effect estimate (95% CI)** | **I2 (95% CI), %** | **No. of studies** | **No. of patients on Intervention** | **No. of patients on Placebo** | **Effect estimate (95% CI)** | **I2 (95% CI), %** |
| **Risperidone** |  |  |  |  |  |  |  |  |  |  |  |  |  |  |  |
| *Efficacy:* |  |  |  |  |  |  |  |  |  |  |  |  |  |  |  |
| PANSS score | 5 | 464 | 331 | SMD=-2.48 (-5.02, 0.07) | 99 (98.6, 99.3) % | 2 | 239 | 163 | SMD=-0.50 (-1.61, 0.61) | 0 (0, 10.5) % | 0 | NA | NA | NA | NA |
| Time to relapse | 4 | 425 | 414 | OR=0.57 (0.14, 2.30) | 87 (68.7, 94.6) % | 3 | 336 | 327 | OR=0.40 (0.22, 0.70) | 0 (0, 89.6) % | 1 | 140 | 135 | **OR=0.33 (0.20, 0.55)** | NA |
| YMRS | 8 | 786 | 731 | **SMD=-3.05 (-5.30, -0.80)** | 99 (98.7, 99.2) % | 6 | 662 | 635 | SMD=-1.22 (-3.10, 0.66) | 98 (97, 98.7) % | 1 | 135 | 133 | **SMD=-0.67 (-0.92, -0.43)** | NA |
| CGI | 7 | 844 | 743 | **SMD=-2.34 (-4.35, -0.33)** | 99 (98.7, 99.3) % | 8 | 910 | 802 | **SMD=-1.00 (-1.94, -0.05)** | 97 (96.0, 99.2) % | 0 | NA | NA | NA | NA |
| *Safety:* |  |  |  |  |  |  |  |  |  |  |  |  |  |  |  |
| TEAEs | 11 | 1533 | 1033 | **RR=1.07 (1.02, 1.12)** | 89 (82.3, 93.2) % | 6 | 856 | 469 | RR=1.10 (0.95, 1.28) | 64 (13, 85.1) % | 2 | 286 | 284 | RR=1.04 (0.88, 1.24) | 73 (0, 93.9) % |
| SAEs | 11 | 1533 | 1033 | RR=0.83 (0.63, 1.10) | 30 (0, 65.5) % | 4 | 552 | 405 | RR=1.19 (0.25, 5.70) | 64 (0, 87.8) % | 2 | 286 | 284 | **RR=0.56 (0.17, 0.84)** | 0% |
| Discontinuation due to AEs | 10 | 1379 | 960 | RR=1.32 (0.92, 1.90) | 0 (0, 62.4) % | 10 | 1343 | 924 | RR=1.16 (0.73, 1.84) | 42 (0, 72.3) % | 2 | 285 | 284 | RR=2.03 (0.52, 7.98) | 0% |
| Death* | 11 | 1455 | 1033 | RD=0 (-0.01, 0.01) | 0 (0, 60.2) % | 8 | 1157 | 750 | RD=0 (-0.01, 0.01) | 0 (0, 67.6) % | 2 | 286 | 284 | RD=0.01 (0, 0.03) | 42% |
| Gynecomastia* | 9 | 1318 | 899 | RD=0 (-0.01, 0.01) | 0 (0, 64.8) % | 0 | NA | NA | NA | NA | 0 | NA | NA | NA | NA |
| Extrapyramidal disorder | 9 | 1196 | 906 | **RD=0.06 (0.04, 0.08)** | 76 (53.9, 87.5) % | 4 | 662 | 438 | RD=0.04 (-0.01, 0.10) | 32 (0, 75.7) % | 0 | NA | NA | NA | NA |
| Tardive dyskinesia | 3 | 506 | 300 | **RD=0.01 (0.001, 0.02)** | 0 (0, 89.6) % | 0 | NA | NA | NA | NA | 0 | NA | NA | NA | NA |
| Akathisia | 6 | 756 | 607 | **RD=0.02 (0.01, 0.03)** | 21 (0, 65.5) % | 1 | 65 | 59 | RD=-0.02 (-0.10, 0.06) | NA | 1 | 132 | 135 | RD=0.03 (-0.01, 0.07) | NA |
| Dystonia | 9 | 1187 | 861 | **RD=0.02 (0.01, 0.03)** | 48 (0, 75.8) % | 0 | NA | NA | NA | NA | 0 | NA | NA | NA | NA |
| Parkinsonism | 4 | 473 | 380 | RD=0.01 (-0.01, 0.03) | 0 (0, 84.7) % | 1 | 106 | 54 | **RD=0.09 (0.01, 0.17)** | NA | 0 | NA | NA | NA | NA |
| Neuroleptic malignant syndrome | 0 | NA | NA | NA | NA | 0 | NA | NA | NA | NA | 0 | NA | NA | NA | NA |
| Sexual Dysfunction | 8 | 1153 | 838 | **RD=0.01 (0.001, 0.01)** | 0 (0, 67.6) % | 0 | NA | NA | NA | NA | 0 | NA | NA | NA | NA |
| Weight increased | 9 | 1298 | 883 | **RD=0.04 (0.02, 0.05)** | 61 (19.2, 81.2) % | 5 | 764 | 499 | RD=0.04 (-0.02, 0.11) | 55 (0, 83.4) % | 2 | 286 | 284 | RD=0.09 (-0.68, 0.81) | 82 (24, 95.7) % |
| Aggression | 5 | 813 | 513 | RD=0 (-0.01, 0.01) | 7 (0, 80.7) % | 1 | 111 | 58 | RD=0.01 (-0.02, 0.04) | NA | 1 | 132 | 135 | RD=0 (-0.01, 0.01) | NA |
| Intentional self-injury | 1 | 72 | 67 | RD=0.01 (-0.02, 0.05) | NA | 0 | NA | NA | NA | NA | 0 | NA | NA | NA | NA |
| Irritability | 6 | 833 | 676 | RD=-0.01 (-0.02, 0) | 0 (0, 74.6) % | 1 | 154 | 149 | RD=-0.02 (-0.05, 0.01) | NA | 0 | NA | NA | NA | NA |
| **Paliperidone** |  |  |  |  |  |  |  |  |  |  |  |  |  |  |  |
| *Efficacy:* |  |  |  |  |  |  |  |  |  |  |  |  |  |  |  |
| PANSS score | 16 | 1653 | 1344 | **SMD=-0.58 (-0.87, -0.29)** | 90 (85.4, 93.1) % | 15 | 1517 | 1202 | **SMD=-0.69 (-1.03, -0.36)** | 92 (84.5, 98.4) % | 3 | 380 | 271 | SMD=-0.58 (-1.52, 0.37) | 82 (44.4, 94.2) % |
| Time to relapse | 4 | 430 | 320 | OR=0.58 (0.06, 6.14) | 94 (87.8, 97.1) % | 2 | 115 | 120 | OR=0.16 (0.09, 0.29) | 78 (4.1, 95) % | 2 | 315 | 200 | OR=1.87 (0.70, 5.04) | 0% |
| YMRS | 5 | 700 | 535 | SMD=-0.64 (-1.49, 0.20) | 95 (91, 97.2) % | 5 | 695 | 529 | **SMD=-0.30 (-0.58, -0.02)** | 68 (41, 92.1) % | 2 | 248 | 160 | SMD=-0.36 (-1.77, 1.06) | 12% |
| CGI | 4 | 520 | 340 | SMD=-0.99 (-2.12, 0.14) | 95 (90.2, 97.5) % | 4 | 520 | 340 | SMD=-1.38 (-4.20, 1.43) | 99 (98.5, 99.3) % | 3 | 379 | 271 | SMD=-0.53 (-1.47, 0.41) | 81 (40.6, 93.9) % |
| *Safety:* |  |  |  |  |  |  |  |  |  |  |  |  |  |  |  |
| TEAEs | 16 | 2775 | 1406 | **RR=1.07 (1.02, 1.13)** | 52 (15.1, 72.9) % | 14 | 2279 | 1234 | RR=1.07 (0.99, 1.16) | 39 (0, 67.6) % | 3 | 484 | 273 | **RR=1.25 (1.05, 1.49)** | 0 (0, 89.6) % |
| SAEs | 16 | 2775 | 1406 | RR=0.90 (0.74, 1.09) | 14 (0, 51.2) % | 8 | 938 | 756 | RR=0.83 (0.46, 1.49) | 0 (0, 67.6) % | 4 | 484 | 380 | RR=0.65 (0.19, 2.25) | 42 (0, 80.5) % |
| Discontinuation due to AEs | 15 | 2620 | 1326 | RR=1.07 (0.66, 1.73) | 46 (1.4, 70.4) % | 15 | 2546 | 1310 | RR=1.07 (0.72, 1.60) | 0 (0, 53.6) % | 4 | 633 | 582 | RR=1.03 (0.40, 2.64) | 29 (0, 73.9) % |
| Death* | 16 | 2677 | 1406 | RD=0 (-0.01, 0) | 0 (0, 52.3) % | 15 | 2312 | 1283 | RD=0 (-0.01, 0) | 0 (0, 53.6) % | 2 | 324 | 315 | RD=0.01 (0, 0.02) | 0% |
| Gynecomastia* | 16 | 2677 | 1406 | RD=0 (0, 0) | 0 (0, 52.3) % | 0 | NA | NA | NA | NA | 0 | NA | NA | NA | NA |
| Extrapyramidal disorders | 15 | 2605 | 1326 | **RD=0.03 (0.02, 0.05)** | 44 (0, 69.5) % | 7 | 1482 | 573 | RD=0.02 (-0.02, 0.07) | 58 (2.9, 81.8) % | 0 | NA | NA | NA | NA |
| Tardive dyskinesia | 10 | 1823 | 894 | RD=-0.00 (-0.01, 0.01) | 0 (0, 62.4) % | 2 | 302 | 212 | RD=0 (-0.01, 0.02) | 0% | 0 | NA | NA | NA | NA |
| Akathisia | 15 | 2569 | 1299 | **RD=0.04 (0.02, 0.05)** | 0 (0, 53.6) % | 8 | 1435 | 672 | **RD=0.03 (0.01, 0.06)** | 28 (0, 67.1) % | 2 | 214 | 122 | RD=0.07 (-0.22, 0.37) | 24% |
| Dystonia | 13 | 2191 | 1182 | **RD=0.02 (0.01, 0.02)** | 31 (0, 64.3) % | 3 | 517 | 193 | RD=0.04 (0, 0.07) | 0 (0, 89.6) % | 1 | 150 | 51 | RD=0.04 (0, 0.08) | NA |
| Parkinsonism | 15 | 2519 | 1326 | **RD=0.03 (0.01, 0.05)** | 62 (33.5, 78.3) % | 0 | NA | NA | NA | NA | 0 | NA | NA | NA | NA |
| Neuroleptic malignant syndrome | 7 | 1030 | 557 | **RD=0.02 (0.01, 0.03)** | 71 (36.8, 86.7) % | 2 | 148 | 118 | RD=0 (-0.02, 0.02) | 0% | 0 | NA | NA | NA | NA |
| Sexual Dysfunction | 16 | 2677 | 1406 | RD=0 (0, 0.01) | 0 (0, 52.3) % | 2 | 222 | 151 | RD=-0.01 (-0.01, 0.03) | 0% | 0 | NA | NA | NA | NA |
| Weight increased | 16 | 2677 | 1406 | **RD=0.02 (0.01, 0.03)** | 45 (1.2, 69.4) % | 4 | 788 | 388 | RD=0 (-0.03, 0.02) | 0 (0, 84.7) % | 2 | 214 | 122 | RD=0.02 (-0.26, 0.31) | 54 (0, 88.7) % |
| Aggression | 16 | 2677 | 1406 | RD=-0.001 (-0.01, 0) | 0 (0, 52.3) % | 1 | 64 | 71 | RD=0.02 (-0.05, 0.08) | NA | 1 | 64 | 71 | RD=-0.01 (-0.05, 0.03) | NA |
| Intentional self-injury | 13 | 2002 | 1136 | RD=-0.001 (-0.01, 0) | 0 (0, 56.6) % | 1 | 64 | 71 | RD=-0.04 (-0.1, 0.02) | NA | 0 | NA | NA | NA | NA |
| Irritability | 15 | 2605 | 1326 | RD=-0.001 (-0.01, 0) | 0 (0, 53.6) % | 0 | NA | NA | NA | NA | 0 | NA | NA | NA | NA |
| **Paliperidone palmitate** |  |  |  |  |  |  |  |  |  |  |  |  |  |  |  |
| *Efficacy:* |  |  |  |  |  |  |  |  |  |  |  |  |  |  |  |
| PANSS score | 8 | 1089 | 1117 | **SMD=-0.43 (-0.56, -0.29)** | 42 (0, 74.4) % | 6 | 803 | 828 | **SMD=-0.43 (-0.62, -0.24)** | 53 (0, 81.2) % | 2 | 317 | 306 | **SMD=-0.56 (-0.89, -0.24)** | 66 (0, 92.3) % |
| Time to relapse | 3 | 367 | 357 | OR=0.55 (0.01, 22.60) | 93 (82.9, 97.1) % | 3 | 367 | 357 | **OR=0.44 (0.30, 0.64)** | 93 (82.9, 97.1) % | 1 | 158 | 164 | **OR=3.16 (1.63, 6.13)** | NA |
| YMRS | 0 | NA | NA | NA | NA | 0 | NA | NA | NA | NA | 0 | NA | NA | NA | NA |
| CGI | 1 | 159 | 142 | **SMD=-0.41 (-0.63, -0.18)** | NA | 0 | NA | NA | NA | NA | 1 | 159 | 142 | **SMD=-0.41 (-0.63, -0.18)** | NA |
| *Safety:* |  |  |  |  |  |  |  |  |  |  |  |  |  |  |  |
| TEAEs | 8 | 2114 | 1192 | RR=1.02 (0.96, 1.07) | 0 (0, 67.6) % | 2 | 160 | 145 | RR=1.03 (0.64, 1.66) | 0 (0, 24) % | 4 | 971 | 643 | RR=1.13 (0.96, 1.32) | 8 (0, 85.9) % |
| SAEs | 8 | 2114 | 1192 | **RR=0.61 (0.43, 0.85)** | 22 (0, 63.9) % | 2 | 160 | 145 | RR=0.35 (0.01, 9.10) | 0 (0, 18.4) % | 5 | 1137 | 801 | **RR=0.54 (0.33, 0.88)** | 14 (0, 82.1) % |
| Discontinuation due to AEs | 8 | 1920 | 1192 | RR=0.75 (0.39, 1.45) | 42 (0, 74.4) % | 6 | 1273 | 865 | RR=0.85 (0.28, 2.57) | 58 (0, 83) % | 5 | 807 | 801 | RR=0.78 (0.57, 1.06) | 0 (0, 79.2) % |
| Death* | 8 | 2114 | 1192 | RD=0 (0, 1) | 0 (0, 67.6) % | 6 | 1792 | 944 | RD=0 (0, 0.01) | 0 (0, 74.6) % | 2 | 214 | 122 | RD=-0.01 (-0.03, 0.02) | 0% |
| Gynecomastia* | 8 | 2114 | 1192 | RD=0 (0, 0.01) | 0 (0, 67.6) % | 0 | NA | NA | NA | NA | 0 | NA | NA | NA | NA |
| Extrapyramidal disorders | 8 | 2114 | 1192 | **RD=0.01 (0.001, 0.02)** | 0 (0, 67.6) % | 2 | 550 | 272 | RD=0.02 (-0.24, 0.29) | 44% | 1 | 159 | 164 | RD=0.05 (-0.01, 0.11) | NA |
| Tardive dyskinesia | 8 | 2114 | 1192 | RD=0 (-0.01, 0) | 0 (0, 67.6) % | 2 | 857 | 367 | RD=0 (-0.01, 0.01) | 0% | 1 | 159 | 164 | RD=-0.01 (-0.02, 0.01) | NA |
| Akathisia | 8 | 2114 | 1192 | RD=0.01 (-0.01, 0.02) | 16 (0, 59) % | 2 | 324 | 315 | RD=0.02 (0, 0.05) | 0% | 4 | 971 | 643 | RD=0.02 (-0.02, 0.05) | 23 (0, 88.2) % |
| Dystonia | 8 | 2114 | 1192 | RD=0.01 (0, 0.02) | 23 (0, 64.6) % | 0 | NA | NA | NA | NA | 1 | 159 | 164 | RD=0.02 (-0.01, 0.05) | NA |
| Parkinsonism | 8 | 2114 | 1192 | **RD=0.01 (0.001, 0.03)** | 32 (0, 69.8) % | 0 | NA | NA | NA | NA | 2 | 323 | 334 | RD=-0.01 (-0.02, 0.01) | 0% |
| Neuroleptic malignant syndrome | 8 | 2114 | 1192 | RD=0 (0, 0.01) | 0 (0, 67.6) % | 0 | NA | NA | NA | NA | 0 | NA | NA | NA | NA |
| Sexual Dysfunction | 8 | 2114 | 1192 | RD=0 (0, 0.01) | 0 (0, 67.6) % | 0 | NA | NA | NA | NA | 0 | NA | NA | NA | NA |
| Weight increased | 8 | 2114 | 1192 | **RD=0.03 (0.01, 0.05)** | 63 (20.4, 82.8) % | 4 | 919 | 645 | **RD=0.04 (0.02, 0.07)** | 0 (0, 84.7) % | 3 | 483 | 479 | RD=0.03 (-0.01, 0.08) | 0 (0, 89.6) % |
| Aggression | 8 | 2114 | 1192 | RD=0 (-0.02, 0.02) | 49 (0, 77.3) % | 0 | NA | NA | NA | NA | 1 | 159 | 164 | RD=0.02 (-0.01, 0.05) | NA |
| Intentional self-injury | 8 | 2114 | 1192 | RD=-0.001 (-0.01, 0) | 0 (0, 67.6) % | 0 | NA | NA | NA | NA | 1 | 159 | 164 | RD=0 (-0.01, 0.01) | NA |
| Irritability | 6 | 1519 | 862 | RD=-0.01 (-0.02, 0) | 0 (0, 74.6) % | 1 | 160 | 145 | RD=-0.01 (-0.04, 0.01) | NA | 2 | 319 | 309 | RD=-0.01 (-0.03, 0) | 0% |

*Analysis was done using the ‘*exact fixed-effect meta-analysis method* *(Tian 2009)’* because many studies reported zero or single events across studies and therefore using traditional meta-analysis methods were considered not reliable enough to synthesis data as the could introduce bias.
