# Supplementary material for: Benefits and harms of Risperidone and Paliperidone for treatment of patients with schizophrenia or bipolar disorder: a meta-analysis involving individual participant data and clinical study reports
Source: BMC Med. 2021 Aug 25;19:195. doi: 10.1186/s12916-021-02062-w (PMC8386072; doi:10.1186/s12916-021-02062-w)
Supplement: Supplementary file 9 — Additional file 9. Table S9 Relative risk and ratio of relative risk between document sources for total AEs and SAEs. [file 12916_2021_2062_MOESM9_ESM.docx]

# Additional file 9: Table S9: Relative risk and ratio of relative risk between document sources for total AEs and SAEs

| **Source** | **CSR** | | | | | **Journal publication** | | | | | **Registry report** | | | | | **Ratio of RRs** | | |
| --- | --- | --- | --- | --- | --- | --- | --- | --- | --- | --- | --- | --- | --- | --- | --- | --- | --- | --- |
| **Treatment arm** | **Treatment** | | **Placebo** | | **Estimate** | **Treatment** | | **Placebo** | | **Estimate** | **Treatment** | | **Placebo** | | **Estimate** | **Source comparison** | | |
| **Outcome** | **Events** | **N** | **Events** | **N** | **RR (95% CI)** | **Events** | **N** | **Events** | **N** | **RR (95% CI)** | **Events** | **N** | **Events** | **N** | **RR (95% CI)** | **CSR vs. Pub** | **CSR vs. Reg** | **Pub vs. Reg** |
| Total AEs | 4434 | 6422 | 2323 | 3631 | **1.05 (1, 1.1)** | 2296 | 6422 | 1142 | 3631 | **1.08 (1.02, 1.15)** | 747 | 6422 | 514 | 3631 | **1.15 (1.04, 1.27)** | 0.97 (0.90, 1.05) | 0.91 (0.81, 1.02) | 0.94 (0.84, 1.06) |
| Total SAEs | 650 | 6422 | 444 | 3631 | **0.75 (0.64, 0.87)** | 82 | 6422 | 81 | 3631 | 0.87 (0.56, 1.35) | 130 | 6422 | 158 | 3631 | **0.57 (0.42, 0.77)** | 0.86 (0.54, 1.37) | 1.32 (0.94, 1.85) | 1.53 (0.89, 2.60) |
|  |  |  |  |  |  |  |  |  |  |  |  |  |  |  |  | **Relative difference*** | | |
|  |  |  |  |  |  |  |  |  |  |  |  |  |  |  |  | **CSR vs. Pub** | **CSR vs. Reg** | **Pub vs. Reg** |
|  |  |  |  |  |  |  |  |  |  |  |  |  |  |  | Total AEs | **1.93 (1.86, 2.00)** | **5.94 (5.54, 6.36)** | **3.07 (2.85, 3.31)** |
|  |  |  |  |  |  |  |  |  |  |  |  |  |  |  | Total SAEs | **7.93 (6.32, 9.95)** | **5 (4.16, 6.02)** | **0.63 (0.48, 0.83)** |

CSR: clinical study report; RRs: relative risks; N: total number of patients in treatment group; CI: confidence interval; AEs: adverse events; SAEs: serious adverse events; Pub: Journal publication; Reg: Registry report

*Relative risk was calculated based on harm caused by any antipsychotic intervention (excluding harm from placebo) and used the total number of patients n=6422 as the denominator.
