# Supplementary material for: Benefits and harms of Risperidone and Paliperidone for treatment of patients with schizophrenia or bipolar disorder: a meta-analysis involving individual participant data and clinical study reports
Source: BMC Med. 2021 Aug 25;19:195. doi: 10.1186/s12916-021-02062-w (PMC8386072; doi:10.1186/s12916-021-02062-w)
Supplement: Supplementary file 10 — Additional file 10. Fig S1 Safety outcome reporting (AEs, SAEs and discontinuations) by study level. [file 12916_2021_2062_MOESM10_ESM.docx]

# Additional file 10: Fig S1: Safety outcome reporting by study level

**Figure 1: Reporting of treatment-emergent adverse events (TEAEs) across all three sources of data**

CSRs: clinical study reports; TEAEs: treatment-emergent adverse events

**Figure 2: Reporting of treatment-emergent serious adverse events (TESAEs) across all three sources of data**

CSRs: clinical study reports; TESAEs: treatment-emergent serious adverse events

**Figure 3: Discontinuations due to an adverse event across each source of data**

CSRs: clinical study reports; AEs: adverse events
