# Supplementary material for: Benefits and harms of Risperidone and Paliperidone for treatment of patients with schizophrenia or bipolar disorder: a meta-analysis involving individual participant data and clinical study reports
Source: BMC Med. 2021 Aug 25;19:195. doi: 10.1186/s12916-021-02062-w (PMC8386072; doi:10.1186/s12916-021-02062-w)
Supplement: Supplementary file 11 — Additional file 11. Table S10 Sensitivity analysis and narrative assessment of cause of death and gynecomastia cases. [file 12916_2021_2062_MOESM11_ESM.docx]

# Additional file 11: Table S10: Sensitivity analysis and narrative assessment of cause of death and gynecomastia cases

**Table 1: Causes of all death**

|  |  | **Treatment** | | **Placebo** | |  |
| --- | --- | --- | --- | --- | --- | --- |
| **Study ID** | **Treatment** | **N** | **Events** | **N** | **Events** | **Cause of death (information collated across all data sources)** |
| RIS-BIP-302 | Risperidone | 72 | 1 | 67 | 1 | Three deaths were reported during the trial: 1 in a patient randomized to adjunctive RLAT (cause of death was hypertensive heart disease, judged to be unrelated to study drug by the investigator) and 2 in patients randomized to adjunctive placebo treatment. |
| RISBIM3003 | Risperidone | 154 | 3 | 149 | 0 | One due to perforated duodenal ulcer and peritonitis (period II), one following an accidental fall, and one due to chemical poisoning and completed suicide (period III) |
| RIS-USA-121 | Risperidone | 302 | 0 | 98 | 1 | Unnatural cause that were not self-inflicted |
| RIS-USA-102 | Risperidone | 52 | 1 | 51 | 0 | Streptococcal pneumonia |
| RIS-INT-69 | Risperidone | 154 | 1 | 140 | 0 | Suicide |
| RIS-USA-239 | Risperidone | 134 | 0 | 125 | 2 | Motor vehicle accident and choking accident |
| RISBMN3001 | Risperidone | 132 | 0 | 135 | 0 | 1 death occurred during screening period |
| R076477-SCH-303 | Paliperidone | 375 | 0 | 126 | 0 | 1 death reported but they were receiving olanzapine |
| R076477-SCH-302 | Paliperidone | 76 | 0 | 38 | 2 | Both deaths recorded as discontinuation, 'grand mal convulsions' and 'status epilepticus’ was recorded as reasons for discontinuation |
| R076477-SCH-301 | Paliperidone | 104 | 0 | 102 | 2 | 1 death due to suicide and other death not explained |
| R076477-BIM-3002 | Paliperidone | 194 | 1 | 105 | 0 | Depression probably related to paliperidone |
| R076477SCH3015 | Paliperidone | 158 | 0 | 80 | 1 | 1 death on placebo group unknown cause |
| R076477-SCH-701 | Paliperidone | 72 | 0 | 80 | 0 | 1 subject died during OL extension with convulsion pulmonary embolism |
| R092670PSY3004 | Paliperidone palmitate | 390 | 1 | 127 | 1 | 2 deaths (1 death in placebo and 100mg paliperidone group respectively) |
| R092670PSY3001 | Paliperidone palmitate | 205 | 0 | 203 | 0 | 3 deaths reported during transition and maintenance phase due to suicide (n=1), accident, fall from window (n=1) and stroke (n=1). But none in the double-blind phase |
| R092670PSY3007 | Paliperidone palmitate | 652 | 1 | 164 | 0 | 1 death on the 150mg group due to cerebrovascular accident |
| R092670SCA3004 | Paliperidone palmitate | 164 | 2 | 170 | 0 | 2 deaths; 1 due to overdose of sleeping pills and another due to coronary artery disease |
| PALM-JPN-4 | Paliperidone palmitate | 159 | 0 | 164 | 1 | 1 death in placebo arm, due to experienced loss of consciousness which resulted in death |
| R092670PSY3012 | Paliperidone palmitate | 160 | 1 | 145 | 0 | 1 death in open label phase (due to toxic megacolon) |
| R076477-SCH-3041 | Paliperidone | 64 | 0 | 71 | 1 | 1 death in placebo group due to suicide |
| R076477-SCH-305 | Paliperidone | 364 | 0 | 123 | 1 | 1 subject in placebo group who withdrew committed suicide later that day! |

**Table 2: Patient narrative for all gynecomastia cases**

|  |  | **Treatment** | | **Placebo** | |  |
| --- | --- | --- | --- | --- | --- | --- |
| **Study ID** | **Treatment** | **N** | **Events** | **N** | **Events** | **Patient narrative** |
| RIS-BIP-302 | Risperidone | 72 | 1 | 67 | 0 | White, male patient aged 21 years, had not recovered in the trial period, probable drug related, consider none-serious and of mild severity |
| R092670PSY3001 | Paliperidone palmitate | 205 | 0 | 203 | 1 | Asian male 42 years old, event possibly related to placebo, considered mild and non-severe |
| R092670PSY3003 | Paliperidone palmitate | 221 | 1 | 135 | 0 | Black male between the ages of 36 to 40 years old, highly likely to be related to treatment, considered moderate severity |
| R092670PSY3012 | Paliperidone palmitate | 160 | 1 | 145 | 0 | Full narrative not reported but was a male patient in the open label phase after receiving paliperidone palmitate |
