# Supplementary material for: Benefits and harms of Risperidone and Paliperidone for treatment of patients with schizophrenia or bipolar disorder: a meta-analysis involving individual participant data and clinical study reports
Source: BMC Med. 2021 Aug 25;19:195. doi: 10.1186/s12916-021-02062-w (PMC8386072; doi:10.1186/s12916-021-02062-w)
Supplement: Supplementary file 12 — Additional file 12. Table S11 Effect estimates based on condition (schizophrenia or bipolar disorder). [file 12916_2021_2062_MOESM12_ESM.docx]

# Additional file 12: Table S11: Effect estimates based on condition (schizophrenia or bipolar disorder)

| **Outcomes** | **CSR** | | **Journal publication** | | **Registry report** | |
| --- | --- | --- | --- | --- | --- | --- |
|  | **Effect estimate (95% CI)** | **I2, %** | **Effect estimate (95% CI)** | **I2, %** | **Effect estimate (95% CI)** | **I2, %** |
| **Schizophrenia** |  |  |  |  |  |  |
| *Efficacy:* |  |  |  |  |  |  |
| PANSS | **SMD=-0.88 (-1.35, -0.41)** | 96% | **SMD=-1.11 (-1.78, -0.43)** | 97% | **SMD=-0.56 (-0.89, -0.24)** | 66% |
| Time to relapse | OR=0.53 (0.18, 1.56) | 92% | **OR=0.32 (0.24, 0.44)** | 89% | OR=2.14 (1.54, 2.98) | 0% |
| YMRS | **SMD=-0.41 (-0.79, -0.02)** | 0% | **SMD=-3.21 (-3.52, -2.90)** | 98% | **SMD=-0.36 (-0.56, -0.15)** | 12% |
| CGI | SMD=-1.35 (-2.74, 0.03) | 98% | **SMD=-3.28 (-5.87, -0.69)** | 99% | **SMD=-0.49 (-0.99, -0.01)** | 73% |
| *Safety:* |  |  |  |  |  |  |
| TEAEs | **RR=1.03 (1.01, 1.07)** | 74% | RR=1.07 (0.99, 1.15) | 38% | **RR=1.15 (1.05, 1.26)** | 0% |
| SAEs | **RR=0.73 (0.61, 0.87)** | 42% | RR=0.79 (0.36, 1.73) | 43% | **RR=0.55 (0.42, 0.73)** | 12% |
| Extrapyramidal disorder | **RD=0.03 (0.01, 0.05)** | 64% | RD=0.03 (0.00, 0.06) | 47% | RD=0.05 (-0.01, 0.11) | NA |
| **Bipolar disorder** |  |  |  |  |  |  |
| *Efficacy:* |  |  |  |  |  |  |
| PANSS | SMD=-0.97 (-4.62, 2.68) | 98% | SMD=-0.08 (-0.36, 0.20) | NA | NA | NA |
| Time to relapse | OR=0.41 (0.03, 5.32) | 19% | **OR=0.40 (0.22, 0.70)** | 0% | **OR=0.33 (0.20, 0.55)** | NA |
| YMRS | **SMD=-2.73 (-4.80, -0.66)** | 99% | **SMD=-0.59 (-0.69, -0.50)** | 98% | **SMD=-0.67 (-0.92, -0.43)** | NA |
| CGI | SMD=-2.25 (-5.07, 0.56) | 99% | **SMD=-0.82 (-0.94, -0.71)** | 98% | NA | NA |
| *Safety:* |  |  |  |  |  |  |
| TEAEs | **RR=1.12 (1.06, 1.19)** | 61% | RR=1.12 (0.95, 1.32) | 59% | RR=1.04 (0.88, 1.24) | 73% |
| SAEs | RR=0.86 (0.64, 1.15) | 0% | RR=0.90 (0.32, 2.51) | 45% | **RR=0.56 (0.38, 0.84)** | 0% |
| Extrapyramidal disorder | **RD=0.03 (0.01, 0.06)** | 40% | RD=0.05 (-0.07, 0.16) | 53% | NA | NA |

CSR: clinical study report; CI: confidence interval; SMD: standardised mean difference; OR: odds ratio; RR: relative risk; RD: risk difference; NA: not applicable; PANSS: Positive and Negative Syndrome Scale; YMRS: Young Mania Rating Scale; CGI-S: Clinical Global Impression rating scales; TEAEs: Treatment-emergent adverse events; SAEs: serious adverse events.
