# Supplementary material for: Benefits and harms of Risperidone and Paliperidone for treatment of patients with schizophrenia or bipolar disorder: a meta-analysis involving individual participant data and clinical study reports
Source: BMC Med. 2021 Aug 25;19:195. doi: 10.1186/s12916-021-02062-w (PMC8386072; doi:10.1186/s12916-021-02062-w)
Supplement: Supplementary file 13 — Additional file 13. Table S12 Effect estimates based on treatment dose for the three outcomes PANSS, AEs and SAEs. [file 12916_2021_2062_MOESM13_ESM.docx]

# Additional file 13: Table S12: Effect estimates based on treatment dose for the three outcomes PANSS, TEAEs and TESAEs

| **Outcomes** |  | **CSR** | | | **Journal publication** | | | **Registry report** | | |
| --- | --- | --- | --- | --- | --- | --- | --- | --- | --- | --- |
|  | **Dose*** | **No. studies** | **Effect estimate (95% CI)** | **I2, %** | **No. studies** | **Effect estimate (95% CI)** | **I2, %** | **No. studies** | **Effect estimate (95% CI)** | **I2, %** |
| **PANSS:** |  |  |  |  |  |  |  |  |  |  |
| Risperidone | ≤ 6 mg | 8 | SMD=-3.69 (-9.78, 2.39) | 99% | 5 | SMD=-4.99 (-15.19, 5.21) | 99% | NA | NA | NA |
| Risperidone | > 6 mg | 2 | SMD=-4.04 (-8.31, 0.23) | 99% | 1 | **SMD=-0.59 (-0.76, -0.42)** | 0% | NA | NA | NA |
| Paliperidone | ≤ 6 mg | 2 | **SMD=-0.41 (-0.79, -0.03)** | 40% | 2 | **SMD=-0.41 (-0.79, -0.03)** | 40% | 1 | SMD=-0.26 (-0.53, 0.01) | NA |
| Paliperidone | > 6 mg | 16 | SMD=-2 (-5.23, 1.23) | 96% | 15 | SMD=-2.55 (-6.02, 0.92) | 98% | 3 | SMD=-0.63 (-1.41, 0.15) | 73% |
| Paliperidone palmitate | < 100 mg | 6 | **SMD=-0.36 (-0.48, -0.25)** | 19% | 5 | **SMD=-0.38 (-0.54, -0.22)** | 39% | NA | NA | NA |
| Paliperidone palmitate | ≥ 100 mg | 7 | **SMD=-0.50 (-0.58, -0.42)** | 0% | 5 | **SMD=-0.51 (-0.63, -0.38)** | 0% | 2 | SMD=-0.56 (-1.28, 0.16) | 0% |
| **TEAEs:** |  |  |  |  |  |  |  |  |  |  |
| Risperidone | ≤ 6 mg | 7 | **RR=1.19 (1.07, 1.32)** | 58% | 3 | **RR=1.23 (1.09, 1.39)** | 0% | NA |  |  |
| Risperidone | > 6 mg | 5 | RR=0.93 (0.81, 1.06) | 81% | 3 | RR=0.99 (0.89, 1.10) | 12% | 1 | RR=1.20 (0.99, 1.44) | NA |
| Paliperidone | ≤ 6 mg | 5 | RR=1.04 (0.91, 1.18) | 43% | 3 | RR=1.13 (0.79, 1.62) | 52% | 1 | **RR=1.27 (1.04, 1.55)** | NA |
| Paliperidone | > 6 mg | 16 | RR=1.08 (0.98, 1.18) | 58% | 14 | **RR=1.09 (1.01, 1.17)** | 40% | 4 | RR=1.10 (0.77, 1.57) | 35% |
| Paliperidone palmitate | < 100 mg | 6 | RR=1 (0.94, 1.08) | 0% | NA | NA | NA | 3 | RR=1.13 (0.82, 1.54) | 31% |
| Paliperidone palmitate | ≥ 100 mg | 5 | RR=0.99 (0.92, 1.07) | 0% | 1 | RR=1.07 (0.89, 1.28) | NA | 2 | **RR=1.16 (1.10, 1.23)** | 0% |
| **TESAEs:** |  |  |  |  |  |  |  |  |  |  |
| Risperidone | ≤ 6 mg | 7 | RR=1.03 (0.84, 1.27) | 0% | 3 | RR=1.85 (0.95, 3.61) | 0% | NA |  |  |
| Risperidone | > 6 mg | 5 | **RR=0.65 (0.50, 0.85)** | 0% | 1 | **RR=0.41 (0.18, 0.90)** | NA | 2 | **RR=0.56 (0.38, 0.84)** | 0% |
| Paliperidone | ≤ 6 mg | 5 | RR=0.89 (0.57, 1.38) | 0% | 1 | RR=1.85 (0.42, 8.10) | 0% | 1 | RR=1.65 (0.62, 4.38) | NA |
| Paliperidone | > 6 mg | 16 | RR=0.79 (0.61, 1.01) | 14% | 8 | RR=0.81 (0.52, 1.27) | 0% | 3 | **RR=0.45 (0.23, 0.89)** | 0% |
| Paliperidone palmitate | < 100 mg | 7 | **RR=0.67 (0.50, 0.90)** | 2% | NA | NA | NA | 3 | **RR=0.54 (0.36, 0.80)** | 0% |
| Paliperidone palmitate | ≥ 100 mg | 5 | **RR=0.63 (0.41, 0.97)** | 27% | 1 | **RR=0.24 (0.08, 0.71)** | NA | 2 | **RR=0.63 (0.43, 0.92)** | 62% |

CSR: clinical study report; CI: confidence interval; SMD: standardised mean difference; OR: odds ratio; RR: relative risk; RD: risk difference; NA: not applicable; PANSS: Positive and Negative Syndrome Scale; TEAEs: Treatment-emergent adverse events; TESAEs: Treatment-emergent serious adverse events.

*Dose thresholds were based on FDA, EMA, and MHRA recommended prescription policy guidelines.
