# Supplementary material for: Benefits and harms of Risperidone and Paliperidone for treatment of patients with schizophrenia or bipolar disorder: a meta-analysis involving individual participant data and clinical study reports
Source: BMC Med. 2021 Aug 25;19:195. doi: 10.1186/s12916-021-02062-w (PMC8386072; doi:10.1186/s12916-021-02062-w)
Supplement: Supplementary file 14 — Additional file 14. Fig S2 Forest plots of all meta-analysis. [file 12916_2021_2062_MOESM14_ESM.docx]

# Additional file 14: Fig S2: forest plot for all meta-analyses

**Total PANSS**

**IPD**

**Risperidone (SMD)**

**Paliperidone (SMD)**

**Paliperidone Palmitate (SMD)**

CSRs

Journal publication

Registry report

**Relapse**

CSRs

Journal publication

Registry report

**CGI-S**

CSRs

Journal publication

Registry report

**YMRS**

CSRs

Publication

Registry report

**AEs**

CSRs

Publication

Registry report

**SAEs**

CSRs

Publication

Registry report

**Discontinue due to AE**

CSRs

Publication

Registry report

**Deaths**

CSRs

Publication

Registry report

**Extrapyramidal disorder**

CSRs

Publication

Registry report

**Tardive dyskinaesia**

CSRs

Publication

Registry report

**Weight increased**

CSRs

Publication

Registry report

**Dystonia**

CSRs

Publication

Registry report

**Akathisia**

CSRs

Publication

Registry report

**Parkinsonism**

CSRs

Publication

Registry report

**Gynecomastia**

CSRs

**Intentional self-injury**

CSRs

Publication

Registry report

**Irritability**

CSRs

Publication

Registry report

**Neuroleptic malignant syndrome**

CSRs

Publication

**Sexual Dysfunction**

CSRs

Publication

**Aggression**

CSRs

Publication

Registry report

**Condition subgroup analysis**

**Total PANSS**

CSRs

Publication

Registry report

**Relapse**

CSRs

Publication

Registry report

**CGI-S**

CSRs

Publication

Registry report

**YMRS**

CSRs

Publication

Registry report

**TEAEs**

CSRs

Publication

Registry report

**TESAEs**

CSRs

Publication

Registry report

**Extrapyramidal disorder**

CSRs

Publication

Registry report

**Dose effect**

**Total PANSS**

CSRs

Publication

Registry report

**TEAEs**

CSRs

Publication

Registry report

**TESAEs**

CSRs

Publication

Registry report
