# Supplementary material for: Benefits and harms of Risperidone and Paliperidone for treatment of patients with schizophrenia or bipolar disorder: a meta-analysis involving individual participant data and clinical study reports
Source: BMC Med. 2021 Aug 25;19:195. doi: 10.1186/s12916-021-02062-w (PMC8386072; doi:10.1186/s12916-021-02062-w)
Supplement: Supplementary file 15 — Additional file 15. Fig S3 – Sensitivity analysis of low RoB studies [file 12916_2021_2062_MOESM15_ESM.docx]

# Additional file 15: Fig S3: Sensitivity analysis of Low RoB studies

**Figure 1: Risk of Bias sensitivity analysis of PANSS score**
